# Mortality by age, gene and gender in carriers of pathogenic mismatch repair gene variants receiving surveillance for early cancer diagnosis and treatment: a report from the prospective Lynch syndrome database

**DOI:** 10.1016/j.eclinm.2023.101909

**Published:** 2023-03-20

**Authors:** Mev Dominguez-Valentin, Saskia Haupt, Toni T. Seppälä, Julian R. Sampson, Lone Sunde, Inge Bernstein, Mark A. Jenkins, Christoph Engel, Stefan Aretz, Maartje Nielsen, Gabriel Capella, Francesc Balaguer, Dafydd Gareth Evans, John Burn, Elke Holinski-Feder, Lucio Bertario, Bernardo Bonanni, Annika Lindblom, Zohar Levi, Finlay Macrae, Ingrid Winship, John-Paul Plazzer, Rolf Sijmons, Luigi Laghi, Adriana Della Valle, Karl Heinimann, Tadeusz Dębniak, Robert Fruscio, Francisco Lopez-Koestner, Karin Alvarez-Valenzuela, Lior H. Katz, Ido Laish, Elez Vainer, Carlos Vaccaro, Dirce Maria Carraro, Kevin Monahan, Elizabeth Half, Aine Stakelum, Des Winter, Rory Kennelly, Nathan Gluck, Harsh Sheth, Naim Abu-Freha, Marc Greenblatt, Benedito Mauro Rossi, Mabel Bohorquez, Giulia Martina Cavestro, Leonardo S. Lino-Silva, Karoline Horisberger, Maria Grazia Tibiletti, Ivana do Nascimento, Huw Thomas, Norma Teresa Rossi, Leandro Apolinário da Silva, Attila Zaránd, Juan Ruiz-Bañobre, Vincent Heuveline, Jukka-Pekka Mecklin, Kirsi Pylvänäinen, Laura Renkonen-Sinisalo, Anna Lepistö, Päivi Peltomäki, Christina Therkildsen, Mia Gebauer Madsen, Stefan Kobbelgaard Burgdorf, John L. Hopper, Aung Ko Win, Robert W. Haile, Noralane Lindor, Steven Gallinger, Loïc Le Marchand, Polly A. Newcomb, Jane Figueiredo, Daniel D. Buchanan, Stephen N. Thibodeau, Magnus von Knebel Doeberitz, Markus Loeffler, Nils Rahner, Evelin Schröck, Verena Steinke-Lange, Wolff Schmiegel, Deepak Vangala, Claudia Perne, Robert Hüneburg, Silke Redler, Reinhard Büttner, Jürgen Weitz, Marta Pineda, Nuria Duenas, Joan Brunet Vidal, Leticia Moreira, Ariadna Sánchez, Eivind Hovig, Sigve Nakken, Kate Green, Fiona Lalloo, James Hill, Emma Crosbie, Miriam Mints, Yael Goldberg, Douglas Tjandra, Sanne W. ten Broeke, Revital Kariv, Guy Rosner, Suresh H. Advani, Lidiya Thomas, Pankaj Shah, Mithun Shah, Florencia Neffa, Patricia Esperon, Walter Pavicic, Giovana Tardin Torrezan, Thiago Bassaneze, Claudia Alejandra Martin, Gabriela Moslein, Pål Moller

**Affiliations:** a Department of Tumor Biology, Institute of Cancer Research, The Norwegian Radium Hospital, 0379, Oslo, Norway; b Engineering Mathematics and Computing Lab (EMCL), Interdisciplinary Center for Scientific Computing (IWR), Heidelberg University, Heidelberg, Germany; c Data Mining and Uncertainty Quantification (DMQ), Heidelberg Institute for Theoretical Studies (HITS), Heidelberg, Germany; d Faculty of Medicine and Health Technology, Tampere University and Tays Cancer Center, Tampere University Hospital, Finland; e Department of Gastrointestinal Surgery, Helsinki University Central Hospital, University of Helsinki, Helsinki, Finland; f Applied Tumor Genomics, Research Program Unit, University of Helsinki, Helsinki, Finland; g Division of Cancer and Genetics, Institute of Medical Genetics, Cardiff University School of Medicine, Heath Park, Cardiff, CF14 4XN, UK; h Department of Clinical Genetics, Aalborg University Hospital, 9000, Aalborg, Denmark; i Department of Biomedicine, Aarhus University, DK-8000, Aarhus, Denmark; j Department of Surgical Gastroenterology, Aalborg University Hospital, Aalborg University, 9100, Aalborg, Denmark; k Department of Clinical Medicine, Aalborg University Hospital, Aalborg University, 9100, Aalborg, Denmark; l Melbourne School of Population and Global Health, Centre for Epidemiology and Biostatistics, The University of Melbourne, Parkville, 3010, Victoria, Australia; m Institute for Medical Informatics, Statistics and Epidemiology, University of Leipzig, 04107, Leipzig, Germany; n Institute of Human Genetics, National Center for Hereditary Tumor Syndromes, Medical Faculty, University Hospital Bonn, University of Bonn, 53127, Bonn, Germany; o Department of Clinical Genetics, Leids Universitair Medisch Centrum, 2300RC, Leiden, the Netherlands; p Hereditary Cancer Program, Institut Català d’Oncologia-IDIBELL, L; Hospitalet de Llobregat, 08908, Barcelona, Spain; q Gastroenterology Department, Hospital Clínic de Barcelona, Centro de Investigación Biomédica en Red de Enfermedades Hepáticas y Digestivas (CIBERehd), Institut d’Investigacions Biomediques August Pi i Sunyer (IDIBAPS), Universitat de Barcelona, Barcelona, Spain; r Manchester Centre for Genomic Medicine, Manchester University NHS Foundation Trust, Manchester, M13 9WL, UK; s Faculty of Medical Sciences, Newcastle University, Newcastle Upon Tyne, NE1 7RU, UK; t Campus Innenstadt, Medizinische Klinik und Poliklinik IV, Klinikum der Universität München, 80336, Munich, Germany; u Center of Medical Genetics, 80335, Munich, Germany; v Division of Cancer Prevention and Genetics, IEO, European Institute of Oncology, Fondazione IRCCS Instituto Nazionale dei Tumori, IRCCS, 20141, Milan, Italy; w Division of Cancer Prevention and Genetics, IEO, European Institute of Oncology IRCCS, 20141, Milan, Italy; x Department of Molecular Medicine and Surgery, Karolinska Institutet, 171 76, Stockholm, Sweden; y Service High Risk GI Cancer Gastroenterology, Department Rabin Medical Center, Israel; z Colorectal Medicine and Genetics, The Royal Melbourne Hospital, Melbourne, Australia; aa Department of Medicine, University of Melbourne, Melbourne, Australia; ab Department of Genetics, University of Groningen, University Medical Center Groningen, Groningen, the Netherlands; ac Department of Medicine and Surgery, Laboratory of Molecular Gastroenterology, IRCCS Humanitas Research Hospital, University of Parma, Parma, Italy; ad Hospital Fuerzas Armadas, Grupo Colaborativo Uruguayo, Investigación de Afecciones Oncológicas Hereditarias (GCU), Montevideo, Uruguay; ae Medical Genetics, Institute for Medical Genetics and Pathology, University Hospital Basel, Switzerland; af Department of Genetics and Pathology, International Hereditary Cancer Center, ul. Unii Lubelskiej 1, 71-252, Szczecin, Poland; ag Department of Medicine and Surgery, University of Milan Bicocca, A.O. San Gerardo, Clinic of Obstetrics and Gynecology, Via Pergolesi 33, Monza (MB), Italy; ah Clínica Universidad de los Andes, Chile; ai Department of Gastroenterology, Hadassah, Medical Center, Faculty of Medicine, Hebrew University of Jerusalem, Israel; aj Hadassah Medical Center, Israel; ak Hereditary Cancer Program (PROCANHE) Hospital Italiano de Buenos Aires, Argentina; al Clinical and Functional Genomics Group, A.C.Camargo Cancer Center, Sao Paulo, Brazil; am Lynch Syndrome & Family Cancer Clinic, St Mark's Hospital, Harrow, HA1 3UJ, London, UK; an Gastrointestinal Cancer Prevention Unit, Gastroenterology Department, Rambam Health Care Campus, Haifa, Israel; ao St Vincent's University Hospital, Ireland; ap Department of Gastroenterology, Tel-Aviv Sourasky Medical Center and Sackler Faculty of Medicine, Tel-Aviv University, Israel; aq Foundation for Research in Genetics and Endocrinology, Institute of Human Genetics, FRIGE House, Ahmedabad, 380015, India; ar Soroka University Medical Center, Ben-Gurion University of the Negev, Beer Sheva, Southern Israel, Israel; as University of Vermont, Larner College of Medicine, Burlington, VT, 05405, USA; at Hospital Sirio Libanes, Sao Paulo, Brazil; au University of Tolima, Tolima, Colombia; av Gastroenterology and Gastrointestinal Endoscopy Unit, Division of Experimental Oncology, IRCCS San Raffaele Scientific Institute, Vita-Salute San Raffaele University, 20132, Milan, Italy; aw Surgical Pathology, Instituto Nacional de Cancerologia, Mexico City, Mexico; ax Department of Visceral and Transplantation Surgery, University Hospital of Zurich, Switzerland; ay Ospedale di Circolo ASST Settelaghi, Centro di Ricerca tumori eredo-familiari, Università dell’Insubria, Varese, Italy; az Universidade Federal de Bahia, Bahia, Brazil; ba St Mark's Hospital, Department of Surgery and Cancer, Imperial College London, London, UK; bb Fundación para el Progreso de la Medicina” y “Sanatorio Allende”, Córdoba, Argentina; bc Hospital Universitário Oswaldo Cruz, Universidade de Pernambuco, Recife, Brazil; bd 1st Department of Surgery, Semmelweis University, Hungary; be Department of Medical Oncology, University Clinical Hospital of Santiago de Compostela (SERGAS); Translational Medical Oncology Group (Oncomet), Health Research Institute of Santiago de Compostela (IDIS); Genomes and Disease, Centre for Research in Molecular Medicine and Chronic Diseases (CiMUS), University of Santiago de Compostela (USC), 15706, Santiago de Compostela, Spain; bf Faculty of Sport and Health Sciences, University of Jyväskylä, Jyväskylä, Finland; bg Department of Education and Science, Central Finland Health Care District, Jyväskylä, Finland; bh Department of Medical and Clinical Genetics, University of Helsinki, Helsinki, Finland; bi The Danish HNPCC Register, Clinical Research Centre, Copenhagen University Hospital, Hvidovre, Denmark; bj Department of Urology, Aarhus University Hospital, Denmark; bk Department of Surgery and Transplantation, Rigshospitalet, Copenhagen University Hospital, Denmark; bl Centre for Epidemiology and Biostatistics, Melbourne School of Population and Global Health, The University of Melbourne, Parkville, Victoria, Australia; bm Department of Medicine, Division of Oncology, Stanford Cancer Institute, Stanford University, USA; bn Department of Health Science Research, Mayo Clinic Arizona, USA; bo Lunenfeld Tanenbaum Research Institute, Mount Sinai Hospital, University of Toronto, Canada; bp University of Hawaii Cancer Center, USA; bq Public Health Sciences Division, Fred Hutchinson Cancer Research Center, Seattle, WA, 98109-1024, USA; br Colorectal Oncogenomics Group, Department of Clinical Pathology, The University of Melbourne, Parkville, Victoria, Australia; bs University of Melbourne Centre for Cancer Research, Victorian Comprehensive Cancer Centre, Parkville, Victoria, Australia; bt Genomic Medicine and Family Cancer Clinic, Royal Melbourne Hospital, Parkville, Victoria, Australia; bu Department of Laboratory Medicine and Pathology, Mayo Clinic, Rochester, MN, 55905, USA; bv Department of Applied Tumour Biology, Institute of Pathology, University Hospital Heidelberg, Heidelberg, Germany; bw Institute of Human Genetics, Medical Faculty and University Hospital Düsseldorf, Heinrich-Heine-University Düsseldorf, Germany; bx National Center for Tumor Diseases (NCT), Partner Site Dresden, Dresden, Germany; by Medizinische Klinik und Poliklinik IV, Campus Innenstadt, Klinikum der Universität München, Munich, Germany; bz Department of Medicine, Knappschaftskrankenhaus, Ruhr-University Bochum, Bochum, Germany; ca Department of Internal Medicine, University Hospital Bonn, Bonn, Germany; cb Institute of Pathology, Faculty of Medicine and University Hospital Cologne, Cologne, Germany; cc Technische Universität Dresden, Dresden, Germany; cd Centre for Bioinformatics, Department of Informatics, University of Oslo, Oslo, Norway; ce Centre for Cancer Cell Reprogramming (CanCell), Institute of Clinical Medicine, Faculty of Medicine, University of Oslo, Oslo, Norway; cf Department of Surgery, Central Manchester University Hospitals NHS Foundation Trust and University of Manchester, London, UK; cg Gynaecological Oncology Research Group, Manchester University NHS Foundation Trust, Manchester, UK; ch Division of Obstetrics and Gyneacology, Department of Women's and Children's Health, Karolinska Institutet, Karolinska University Hospital, Solna, Stockholm, Sweden; ci Head Adult Genetic Service, Raphael Recanati Genetic Institute, Rabin Medical Center–Beilinson Hospital, Petach Tikva, Israel; cj Sushrut Hospital and Research Centre, Mumbai, India; ck Zydus Cancer Centre, Ahmedabad, India; cl Instituto de Medicina Traslacional e Ingenieria Biomedica (IMTIB), CONICET IU, Hospital Italiano de Buenos Aires, Buenos Aires, 94, Argentina; cm Hospital Privado Universiatrio de Córdoba, Cordoba, Argentina; cn Surgical Center for Hereditary Tumors, Ev. Bethesda Khs Duisburg, University Witten-Herdecke, Herdecke, Germany; co Department of Medicine, Melbourne University, Melbourne, Australia; cp Programa Cáncer Heredo Familiar, Santiago, Chile; cq Depart-ment of Surgery, Universitätsmedizin Mainz, Germany; cr SEQUIPE, Recife, Brazil; cs Centro de Investigación Biomédica en Red Cáncer (CIBERONC), 28029, Madrid, Spain; ct Department of Surgery, Central Finland Health Care District, Jyväskylä, Finland; cu Cooperation Unit Applied Tumour Biology, German Cancer Research Center (DKFZ), Heidelberg, Germany; cv German Cancer Consortium (DKTK) Dresden and German Cancer Research Center (DKFZ) Heidelberg, Heidelberg, Germany; cw Institute for Clinical Genetics, Faculty of Medicine and University Hospital Carl Gustav Carus, TU Dresden, Dresden, Germany; cx Hereditary Cancer Syndrome Center Dresden, Faculty of Medicine and University Hospital Carl Gustav Carus, TU Dresden, Dresden, Germany; cy MGZ - Medical Genetics Center, Munich, Germany; cz Division of Cancer Sciences, University of Manchester, Manchester, UK

**Keywords:** Mortality, Survival, Lynch syndrome, Cancer risk, *MLH1*, *MSH2*, *MSH6*, *PMS2*, Prospective study

## Abstract

**Background:**

The Prospective Lynch Syndrome Database (PLSD) collates information on carriers of pathogenic or likely pathogenic MMR variants (*path_MMR*) who are receiving medical follow-up, including colonoscopy surveillance, which aims to the achieve early diagnosis and treatment of cancers. Here we use the most recent PLSD cohort that is larger and has wider geographical representation than previous versions, allowing us to present mortality as an outcome, and median ages at cancer diagnoses for the first time.

**Methods:**

The PLSD is a prospective observational study without a control group that was designed in 2012 and updated up to October 2022. Data for 8500 carriers of *path_MMR* variants from 25 countries were included, providing 71,713 years of follow up. Cumulative cancer incidences at 65 years of age were combined with 10-year crude survival following cancer, to derive estimates of mortality up to 75 years of age by organ, gene, and gender.

**Findings:**

Gynaecological cancers were more frequent than colorectal cancers in *path_MSH2, path_MSH6 and path_PMS2* carriers [cumulative incidence: 53.3%, 49.6% and 23.3% at 75 years, respectively]. Endometrial, colon and ovarian cancer had low mortality [8%, 13% and 15%, respectively] and prostate cancers were frequent in male *path_MSH2* carriers [cumulative incidence: 39.7% at 75 years]. Pancreatic, brain, biliary tract and ureter and kidney and urinary bladder cancers were associated with high mortality [83%, 66%, 58%, 27%, and 29%, respectively]. Among *path_MMR* carriers undergoing colonoscopy surveillance, particularly *path_MSH2* carriers, more deaths followed non-colorectal Lynch syndrome cancers than colorectal cancers.

**Interpretation:**

In *path_MMR* carriers undergoing colonoscopy surveillance, non-colorectal Lynch syndrome cancers were associated with more deaths than were colorectal cancers. Reducing deaths from non-colorectal cancers presents a key challenge in contemporary medical care in Lynch syndrome.

**Funding:**

We acknowledge funding from the 10.13039/100008730Norwegian Cancer Society, contract 194751-2017.

**Research in context**

**Evidence before this study**

We searched PubMed up to October, 2022 for articles in English published using the search terms “Lynch syndrome and cancer risk”, “Lynch syndrome and survival”, “Lynch syndrome and mortality”, “extra colonic Lynch syndrome tumor”, “Lynch syndrome and colorectal cancer incidence” and “surveillance and Lynch syndrome” in the title or abstract. However, reduction of colorectal cancer incidence by colonoscopy surveillance has not been documented and there are limited data regarding risks for other cancer types and the effectiveness of wider cancer surveillance in individuals with Lynch syndrome. Previously, outcomes from interventions including colonoscopy have been reported as cancer incidences, not survival.

**Added value of this study**

In carriers of MMR variants undergoing colonoscopy surveillance, colorectal cancer was frequent but associated with low mortality while some other cancers, notably bile duct, pancreas and brain, were associated with high mortality and more deaths followed non-colorectal than colorectal cancers.

**Implications of all the available evidence**

Prevention and treatment of non-colorectal cancers should be prioritised to further reduce mortality in *path_MMR* carriers.

## Introduction

Lynch syndrome (LS) is caused by pathogenic variants in any of the four mismatch repair genes, *MLH1*, *MSH2*, *MSH6* or *PMS2* or by deletion of the 3′end of *EPCAM* (TACSTD1) which results in hypermethylation of the *MSH2* promoter (*path_MMR*).^1^ Colonoscopy with polypectomy has been advocated to prevent colorectal cancer (CRC) in *path_MMR* carriers^2^; but several reports have found high CRC incidence in *path_MMR* carriers despite surveillance colonoscopy, as well as high gynaecological cancer incidence.^3^, ^4^, ^5^, ^6^, ^7^, ^8^ The efficacy of surveillance for non-colorectal cancers in LS is not well evidenced.^9^ Survival following cancer diagnosis has been reported,^4^ guidelines for clinical interventions have been issued^10^ and the extent to which management practices align with research findings and with the guidelines based upon them has been discussed.^11^ The main goal for intervention in a person with an inherited cancer risk is to prevent premature death,^12^ but success in achieving this has been difficult to measure in LS, and the reports mentioned above have focused largely on cancer incidence as a surrogate endpoint for survival.

Recently, the USA National Comprehensive Cancer Network (NCCN) guidelines described the gene- and organ-specific cumulative cancer risks in *path_MMR* carriers based on various data sources, including the Prospective Lynch Syndrome Database (PLSD).^13^ However, when advocating colonoscopy for the prevention of CRC, NCCN did not acknowledge that the CRC incidences reported by PLSD were determined in individuals undergoing colonoscopy surveillance. It was also stated that the incidences of some other cancers had not been reported in the literature and the average ages at diagnosis of cancers were described without indicating how they were obtained.

There is a lack of evidence that surveillance prevents extracolonic cancers, but current guidelines do include surveillance recommendations for some of the many extracolonic cancers associated with LS.^10^^,^^13^, ^14^, ^15^, ^16^ There is limited information on CRC and extracolonic cancer mortality in *path_MMR* carriers who receive colonoscopy surveillance. Providing new data on mortality was the focus of this study.

The updated version of PLSD upon which the current study is based includes 71,713 prospective follow-up years, allowing us for the first time to present mortality outcomes by gene and gender, for each organ in which LS-associated cancers occur. We also present data that will help to fill the knowledge gaps in the recent NCCN guidelines when they are next updated, including the median age at cancer diagnosis in each organ by gene and gender.

## Methods

### The PLSD design

The PLSD is a prospective observational study without a control group that was designed in 2012 and that provides an aggregated compilation of combined genetic and clinical information from all contributors up to October 2022. The eligibility criteria include *path_MMR* carriers with or without a previous cancer who are aged 25 years or older on the day of their first prospectively planned and completed surveillance colonoscopy.^4^, ^5^, ^6^, ^7^, ^8^^,^^10^^,^^11^^,^^17^, ^18^, ^19^ Cancers are grouped by the three first positions in the ICD9 classification system. This fails to identify sebaceous gland cancers. Osteosarcomas are recognised as part of LS,^20^ but were found too infrequently to be included in the presentation of cancer incidences. In this study, cancers in the following organs are denoted as LS cancers: colon, rectum, endometrium, ovary, small intestine, bile duct, pancreas, stomach, prostate, urinary bladder, ureter, brain and osteosarcoma. Adenocarcinomas share some phenotypic characteristics, and there is a small possibility that a subsequent cancer might have been a recurrence from a previous cancer in the same or another organ. However, local recurrences are usually clinically distinguished from metachronous primaries. The *path_MMR* carriers in the current study were followed up with colonoscopy and gynaecological surveillance according to local implementation of international recommendations. The gynaecological follow-up carried out by PLSD contributing centres has been reported previously and is not evidence-based.^4^^,^^17^

### Statistical analysis

Annual incidence rates in 5-year cohorts by gene and gender for cancer in each organ and in groups of organs were calculated in MySQL80©.

Overall survival was estimated using the Nelson-Aalen algorithm in R.^21^ Categorization of carriers as dead or alive was made at last observation and was made for all carriers. Crude mortality at 75 years of age following cancer in specific organs was calculated as cumulative incidence at 65 years for cancer in each organ multiplied by (1–10-year survival) following cancer diagnosis in that organ. The reported mortality is an empirical observation which includes no assumptions, and includes death from any cause, including synchronous and metachronous cancers associated with LS. Possible overdiagnosis of colon cancer due to colonoscopy^21^ was adjusted for when calculating survival: incidence and survival are to be measured simultaneously when combined like this. PLSD incidences should not be compared with survival measured in other ways. We are not aware of any studies to measure mortality by other means and to our knowledge there is no previous report on mortality as an outcome of screening for early cancer diagnosis in LS. Confounders to our method for estimating survival include time-trends in treatments that may reduce mortality, which this report did not consider. The point estimates for *path_PMS2* carriers have wide confidence intervals because of the low number of carriers and follow-up years collated by the PLSD.

Cancers detected prospectively were scored as the first tumor in each organ in carriers who had not had cancer in that organ before or at inclusion (ignoring prospectively diagnosed cancers in other organs and excluding previous cancers and prevalent cancers identified at inclusion). No synchronous or subsequent cancer in the same organ was scored as an event. Cumulative risk for cancer was set to zero at age 25 years, and annual incidence rates (AIRs) for five-year cohorts from 25 to 75 years of age were calculated as the starting point for further calculations in R© (version 4.2.0). In the current report, the cumulative incidences (risks) and their 95% confidence intervals (CIs) were calculated using Nelson-Aalen estimates with an underlying Poisson distribution.

For calculating the median age of cancer diagnosis, the risk has to be conditioned on those patients who developed any cancer during their lifetime. For this, the conditional risk was computed by dividing the risk estimate in each five-year age cohort by the lifetime risk (approximated by the risk at 75 years of age), mapping the risk on the interval [0%; 100%]. The corresponding conditional 95% CIs were computed accordingly, conditioned on the life-time risk and truncated to the interval [0%; 100%]. We then performed a piecewise linear interpolation of the conditional risk and conditional 95% CIs for the five-year age cohorts to determine the median age of cancer onset. The latter corresponds to the age at which the interpolated conditional risk hits the 50% conditional risk limit. The same intersection calculations were performed for the corresponding 95% confidence intervals.

### Ethics statement

The study adhered to the principles set out in the Declaration of Helsinki. It was approved by the Norwegian Data Protection Authority (reference 2001/2988-2) and the Ethics Committee (reference S-02030). Genetic testing was performed with informed consent according to local and national requirements and all reporting centres exported only de-identified data to PLSD. Patients had been followed up prospectively according to international and local clinical guidelines, as previously described.^3^, ^4^, ^5^, ^6^, ^7^^,^^17^, ^18^, ^19^^,^^22^

### Role of the funding source

The funding body had no role in the design of the study and collection, analysis, and interpretation of data and in writing the manuscript. MD-V and PM had access to dataset and all authors contributed data to the PLSD and reviewed and approved the manuscript. All authors have read and agreed to the final version of the manuscript. Version. All authors had full access to all the data in the study and accept responsibility for the decision to submit for publication.

## Results

### Characteristics of the PLSD patients, follow-up years, gene, gender, and country

In addition to the 6350 carriers included in our previous report,^4^ data from 2150 new *path_MMR* carriers were provided by 18 new contributing centres and by previously contributing centres that provided information on newly recruited carriers. In total, 25 countries in five continents (Supplementary Table S1) were represented in the current PLSD dataset that comprised 8500 *path_MMR* carriers (4588 females and 3912 males) with a mean age of 42.5 and 43.6 years for *path_MLH1/MSH2* carriers compared to 48.3 and 49.9 years for p*ath_MSH6/PMS2* carriers at inclusion. They were followed up with surveillance colonoscopy and provided 71,713 prospective observation years, with a mean follow-up time of 8.4 years.

When stratified by gene, there were 3171 (37.3%) *path_MSH2* carriers, 3131 (36.8%) *path_MLH1*, 1649 *path_MSH6* (19.4%) and 549 (6.5%) *path_PMS2* carriers in the study. Supplementary Table S1 describes the numbers of patients, follow-up years and age at inclusion, stratified by gene, gender, and country.

During prospective observation, 1853 first cancers in any organ were diagnosed (Supplementary Table S2) of which 1436 (77.5%) were LS-associated cancers (stomach, small intestine, biliary tract, pancreas, colon, rectum, endometrium, ovaries, osteosarcoma, prostate, brain, urinary bladder and ureter). Cancers of the colon (n = 481, 26% of all cancers), endometrium (n = 237, 12.8%), skin (n = 155, 8.4%), and rectum (n = 137, 7.4%) were most frequent, but skin cancers were not reported consistently. In Supplementary Tables S3–S6, we also present the incidence for breast cancers. Although this cancer is mentioned in the NCCN guidelines,^13^ we do not consider breast cancer to be a part of LS and hence do not discuss the findings.^23^

### Survival and mortality

Ten-year crude survival after cancer of the colon that occurred before 65 years of age was 87% and 72% after rectal, 92% after endometrial, 85% after ovarian, 73% after upper urinary tract, 71% after urinary bladder, 76% after prostate, 42% after bile duct, 63% after stomach, 70% after small bowel, 17% after pancreas and 34% after brain cancers. Five- and 10-year survival are detailed in Supplementary Table S7.

**Fig. 1:**
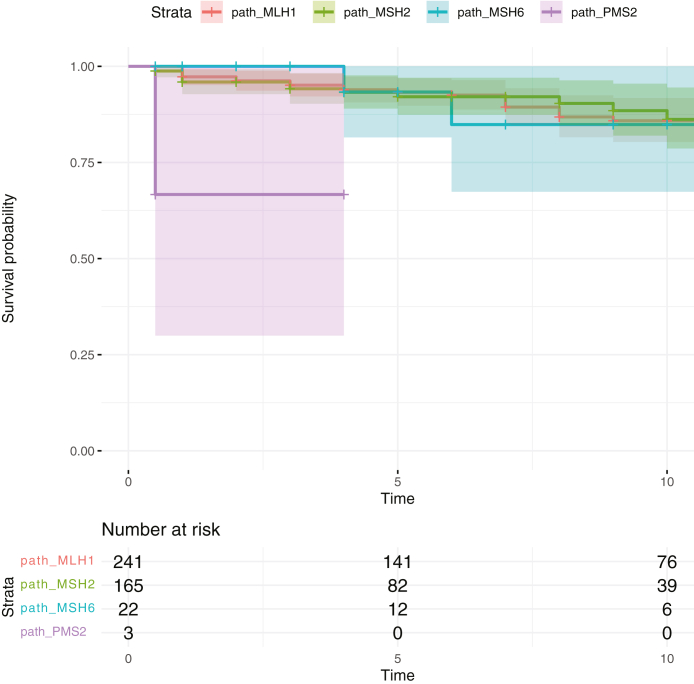
Crude survival (%) in *path_MLH1, path_MSH2, path_MSH6* and *path_PMS2* carriers subjected to colonoscopy surveillance after colon cancer diagnosed before age of 65 years. The dark line showed the survival probability, and the bars showed the 95% confidence interval for each *path_MMR* carrier: *path_MLH1* (orange), *path_MSH2* (green), *path_MSH6* (blue) and *path_PMS2* (purple). Categorization of carriers as dead or alive was made at last observation and was made for all carriers.

**Fig. 2:**
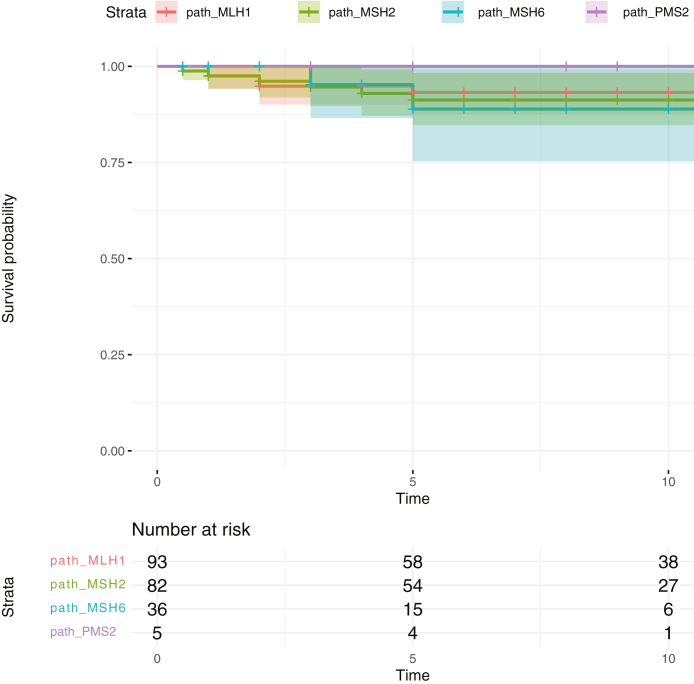
Crude survival (%) in *path_MLH1, path_MSH2*, *path_MSH6* and *path_PMS2* carriers subjected to colonoscopy surveillance after endometrial cancer diagnosed before age of 65 years. The dark line showed the survival probability, and the bars showed the 95% confidence interval for each *path_MMR* carrier: *path_MLH1* (orange), *path_MSH2* (green), *path_MSH6* (blue) and *path_PMS2* (purple). Categorization of carriers as dead or alive was made at last observation and was made for all carriers.

Figs. 1 and 2 illustrate that there were no significant differences by gene in the 10-year survival after colon or endometrial cancer. For colon cancer this was 86% [80%–92%] in *path_MLH1,* 89% [82%–96%] in *path_MSH2* and 85% [67%–100%] in *path_MSH6* carriers and for endometrial cancer: 93% [88%–99%]*,* 91% [85%–98%] and 89% [75%–100%], respectively. Supplementary Figs. S1–S10 show the 10-year survival by gene for rectal and extracolonic cancers, including ovarian, ureter and kidney, urinary bladder, prostate, stomach, small bowel, biliary tract, pancreas, and brain cancers.

Mortality by organ, gene and gender at 75 years of age is presented in Table 1. As shown, for male *path_MLH1* and *path_MSH6* carriers, mortality was similar after CRC compared to mortality after non-CRC cancers. *Path_MSH2* carriers of both genders and female *path_MSH6* carriers had more deaths after non-CRC. Importantly, the combined incidences of deaths after colon and rectal cancer for all *path_MMR* carriers comprised less than half of the total deaths following any LS cancer. Counting numbers (not incidences) of deaths in the total series, deaths following CRC accounted for less than half of all deaths following an LS cancer (n = 76, 36%) (Table 2). Gyneacological cancer (n = 31, 14.8%), ureter and kidney (n = 16, 7.7%), stomach cancer (n = 16, 7.7%) and pancreas (n = 14, 6.7%) were the other cancers associated with a high number of deaths (Table 2).

**Table 1:** Mortality by cancer, gene and gender at 75 years in *path_MMR* carriers: mortality at 75 years was calculated as cumulative incidence at 65 years with [95% confidence intervals] multiplied by (1 – ten years survival) following cancer in that organ.

Cancer type	Pathogenic variants	10-year survival	Males		Females	
			Cumulative incidence 65 years	Mortality 75 years	Cumulative incidence 65 years	Mortality 75 years
Colon	*path_MLH1*	87%	48.4% [42.4–54.8]	6%	36.3% [31.0–42.3]	5%
	*path_MSH2*		41.5% [34.8–48.8]	5%	29.8% [24.6–35.8]	4%
	*path_MSH6*		12.7% [6.8–23.1]	2%	10.1% [5.8–17.1]	1%
	*path_PMS2*		9.5% [2.5–32.9]	1%	2.8% [0.4–18.2]	0%
Rectum	*path_MLH1*	72%	6.0% [3.8–9.3]	2%	4.6% [2.9–7.3]	1%
	*path_MSH2*		12.6% [9.2–17.3]	4%	7.6% [5.1–11.1]	2%
	*path_MSH6*		5.1% [2.3–11.1]	1%	3.9% [1.8–8.6]	1%
	*path_PMS2*		0% [NA]	0%	2.2% [0.3–14.6]	1%
Endometrium	*path_MLH1*	92%	na		31.7% [26.5–37.7]	3%
	*path_MSH2*				37.6% [31.3–44.8]	3%
	*path_MSH6*				32.1% [24.2–41.7]	3%
	*path_PMS2*				12.7% [5.5–27.9]	1%
Ovary	*path_MLH1*	85%	na		8.0% [5.3–12.0]	1%
	*path_MSH2*				10.6% [7.2–15.6]	2%
	*path_MSH6*				2.9% [0.9–8.7]	0%
	*path_PMS2*				2.5% [0.4–16.3]	0%
Stomach	*path_MLH1*	63%	2.8% [1.5–5.2]	1%	2.0% [1.0–4.2]	1%
	*path_MSH2*		4.3% [2.5–7.6]	2%	2.6% [1.4–5.0]	1%
	*path_MSH6*		0.7% [0.1–4.9]	0%	0.7% [0.1–4.7]	0%
	*path_PMS2*		2.7% [0.4–17.5]	1%	0% [NA]	0%
Small intestine	*path_MLH1*	70%	4.4% [2.6–7.2]	1%	2.5% [1.3–4.6]	1%
	*path_MSH2*		4.5% [2.6–7.6]	1%	3.2% [1.8–5.6]	1%
	*path_MSH6*		0.7% [0.1–4.8]	0%	0.6% [0.1–4.0]	0%
	*path_PMS2*		3.3% [0.5–21.3]	1%	2.1% [0.3–14.0]	1%
Bile duct	*path_MLH1*	42%	2.9% [1.5–5.6]	2%	1.5% [0.7–3.3]	1%
	*path_MSH2*		1.0% [0.3–3.2]	1%	0.8% [0.3–2.4]	0%
	*path_MSH6*		0% [NA]	0%	0% [NA]	0%
	*path_PMS2*		0% [NA]	0%	0% [NA]	0%
Pancreas	*path_MLH1*	17%	1.1% [0.4–2.9]	1%	1.9% [0.9–4.0]	2%
	*path_MSH2*		1.4% [0.5–3.7]	1%	1.2% [0.5–3.3]	1%
	*path_MSH6*		0% [NA]	0%	0.7% [0.1–4.8]	1%
	*path_PMS2*		0% [NA]	0%	0% [NA]	0%
Ureter/kidney	*path_MLH1*	73%	2.5% [1.3–5.1]	1%	1.7% [0.8–3.8]	0%
	*path_MSH2*		11.5% [8.2–16.0]	3%	9.7% [6.9–13.5]	3%
	*path_MSH6*		1.4% [0.3–5.4]	0%	3.2% [1.3–7.4]	1%
	*path_PMS2*		0% [NA]	0%	0% [NA]	0%
Urinary bladder	*path_MLH1*	71%	3.3% [1.8–6.1]	1%	1.3% [0.6–3.2]	0%
	*path_MSH2*		5.9% [3.7–9.4]	2%	4.7% [2.8–7.7]	1%
	*path_MSH6*		3.0% [1.1–7.9]	1%	1.8% [0.6–5.6]	1%
	*path_PMS2*		0% [NA]	0%	0% [NA]	0%
Prostate	*path_MLH1*	76%	5.3% [3.2–8.7]	1%	na	
	*path_MSH2*		10.6% [7.5–15.0]	3%		
	*path_MSH6*		3.0% [1.1–7.7]	1%		
	*path_PMS2*		3.3% [0.5–21.5]	1%		
Brain	*path_MLH1*	34%	0% [NA]	0%	0.9% [0.3–2.4]	1%
	*path_MSH2*		3.3% [1.7–6.3]	2%	1.4% [0.5–3.8]	1%
	*path_MSH6*		0.8% [0.1–5.3]	1%	1.2% [0.3–4.6]	1%
	*path_PMS2*		0% [NA]	0%	7.3% [1.1–41.6]	5%^a^

Mortality was calculated as cumulative incidence at 65 years of age multiplied by (1–10 years survival) from Supplementary Table S4.

a Caused by only one case at young age, which is not significantly different from zero, na: not applicable.

**Table 2:** Numbers of deaths by cancer type and *path_MMR* variant.

Cancer	*path_MMR*	n	n deaths
Colon	*path_MLH1*	241	26
	*path_MSH2*	165	20
	*path_MSH6*	22	3
	*path_PMS2*	3	1
Endometrial	*path_MLH1*	93	11
	*path_MSH2*	82	11
	*path_MSH6*	36	2
	*path_PMS2*	5	0
Rectal	*path_MLH1*	36	8
	*path_MSH2*	59	15
	*path_MSH6*	12	3
	*path_PMS2*	1	0
Ovarian	*path_MLH1*	23	2
	*path_MSH2*	25	5
	*path_MSH6*	3	0
	*path_PMS2*	1	0
Ureter and kidney	*path_MLH1*	14	5
	*path_MSH2*	64	11
	*path_MSH6*	7	0
	*path_PMS2*	0	0
Urinary bladder	*path_MLH1*	15	3
	*path_MSH2*	32	8
	*path_MSH6*	7	0
	*path_PMS2*	0	0
Prostate	*path_MLH1*	15	3
	*path_MSH2*	30	6
	*path_MSH6*	4	0
	*path_PMS2*	1	0
Stomach	*path_MLH1*	17	10
	*path_MSH2*	21	5
	*path_MSH6*	2	1
	*path_PMS2*	1	0
Small bowel	*path_MLH1*	25	7
	*path_MSH2*	24	5
	*path_MSH6*	2	0
	*path_PMS2*	2	0
Billiary tract	*path_MLH1*	15	9
	*path_MSH2*	6	2
	*path_MSH6*	0	0
	*path_PMS2*	0	0
Pancreas	*path_MLH1*	11	10
	*path_MSH2*	8	4
	*path_MSH6*	1	0
	*path_PMS2*	0	0
Brain	*path_MLH1*	4	2
	*path_MSH2*	13	9
	*path_MSH6*	3	1
	*path_PMS2*	1	1

### Median age of cancer diagnosis and cumulative incidences of cancers by age, gene, gender, and organ

The median ages of cancer diagnosis and the cumulative incidences of cancers in *path_ MLH1*, *path_MSH2, path_MSH6* and *path_PMS2* carriers in different organs by age, gene and gender are given in Table 3 and Supplementary Tables S3–S6, respectively. We used the same format as the 2021 NCCN report^13^ and present LS-associated cancers only, based on previous PLSD reports but excluding osteosarcoma (the 11 cases found were insufficient for statistical calculations). Incidences of groups of cancers such as urinary tract cancers, endometrial or ovarian cancers and upper gastrointestinal tract cancers are also given.

**Table 3:** Median age of onset for cancer in specific organs by age and gender for *path_MMR* carriers.

Site	Median age of onset for *path_MLH1* carriers		Median age of onset for *path_MSH2* carriers		Median age of onset for *path_MSH6* carriers		Median age of onset for *path_PMS2* carriers	
	Males	Females	Males	Females	Males	Females	Males	Females
Any organ	48.4 [45.8–50.9]	51.3 [49.1–53.3]	53.6 [51.0–56.0]	50.6 [48.1–52.9]	55.8 [NA–64.7]	57.5 [53.0–61.7]	59.6 [NA–73.7]	63.8 [NA]
Colon^a^	47.8 [44.8–50.6]	55.5 [51.84–59.2]	53.6 [50.4–58.0]	55.7 [49.3 61.1]	57.1 [NA–73.0]	60.8 [41.0–67.4]	70.5 [NA]	66.1 [NA]
Sigmoid/Rectum^a^	62.5 [50.2–70.8]	59.3 [46.9–69.2]	57.6 [49.9–64.8]	63.6 [55.0–67.4]	61.3 [NA]	61.2 [NA]	NA	62.5 [NA]
Colorectal^a^	47.4 [44.3–50.4]	54.6 [51.0–58.5]	53.9 [50.0–57.5]	56.2 [50.3–61.3]	55.1 [NA–73.3]	60.1 [42.2–67.1]	71.3 [NA]	66.1 [NA]
Endometrium		51.7 [49.7–53.6]		51.7 [49.4–53.9]		60.0 [55.5–64.1]		61.2 [NA]
Ovary		49.0 [42.1–55.9]		47.4 [42.8–60.8]		65.5 [NA]		57.5 [NA]
Endometrium/ovary		51.2 [49.1–53.0]		50.4 [48.4–52.6]		59.9 [55.5–64.2]		59.5 [NA]
CRC/Endometrium/ovary cancer		51.4 [49.5–53.2]		51.0 [48.7–53.5]		57.7 [50.6–62.6]		64.4 [NA]
Urine bladder	63.0 [56.2–73.5]	68.8 [58.8–74.4]	67.4 [58.7–72.6]	65.1 [54.1–71.9]	71.2 [NA–74.7]	61.1 [NA]	NA	NA
Ureter/kidney	63.9 [50.6–73.7]	63.6 [NA]	61.1 [56.7–64.6]	65.1 [61.6–69.3]	65.7 [NA]	62.0 [NA]	72.5 [NA]	NA
Kidney, ureter and/or urine bladder	62.9 [57.1–71.3]	67.5 [59.4–73.0]	62.5 [59.0–67.4]	64.3 [61.6–68.1]	70.6 [NA–74.2]	61.8 [NA -68.6]	72.5 [NA]	NA
Kidney, ureter and/or urine bladder/prostate	67.5 [62.5–71.4]		62.6 [59.8–65.5]		68.8 [59.2–73.0]		70.9 [NA]	
Stomach	68.1 [59.0–72.7]	65.7 [56.6–74.1]	64.3 [57.46–73.35]	61.9 [NA-73.7]	52.5 [NA]	62.5 [NA]	57.5 [NA]	NA
Small bowel	63.5 [54.0–70.1]	53.8 [NA-71.0]	59.8 [52.6–67.6]	54.2 [NA -66.2]	68.8 [NA]	57.5 [NA]	47.5 [NA]	62.5 [NA]
Pancreas	67.0 [NA]	64.7 [NA -74.8]	65.7 [NA]	66.8 [NA]	72.5 [NA]	66.4 [NA]	NA	NA
Bile duct/gall bladder	62.2 [48.0–68.7]	53.4 [NA]	69.9 [60.2-NA]	71.3 [NA]	NA	NA	NA	NA
Prostate	70.2 [63.7–72.6]		66.2 [62.0–70.0]		65.9 [NA–74.9]		47.5 [NA]	
Breast		57.7 [52.6–63.6]		60.7 [53.5–65.9]		62.7 [51.7–72.4]		70.2 [NA]
Brain	67.5 [NA]	49.9 [NA]	70.0 [43.0–74.6]	52.2 [NA]	62.5 [NA]	54.9 [NA]	NA	NA

NA: occurs for the median age of onset or the confidence limits if the corresponding conditional cumulative incidence estimates always are above or below 50%.

a Following intervention with surveillance colonoscopy as described in text.

Median ages at cancer diagnoses by gene, organ, and gender have not been reported before in LS. We found a younger median age at diagnosis for cancers in *path_MSH2* carriers than in *path_MLH1* carriers, with the exception of CRC, urinary bladder, bile duct/gall bladder and brain cancers. An older median age at diagnosis was observed for cancers in *path_MSH6* and *path_PMS2* carriers.

The highest cumulative incidences of CRC before 50 years of age were observed for *path_MLH1* and *path_MSH2* carriers. Carriers of *path_MSH6* had substantially lower CRC incidence and the cancers occurred predominantly after 50 years of age. However, a 2.7% [0.4–17.7] cumulative incidence of CRC in male *path_MSH6* carriers at 30 years was observed compared to none in the previous PLSD report.^4^ Endometrial cancers were characterised by onset before 50 years of age in *path_MLH1, path_MSH2* and *path_MSH6* carriers. Ovarian cancers started to occur after 40 years of age, most frequently in *path_MSH2* followed by *path_MLH1* and *path_MSH6* carriers. *Path_MSH2* carriers were at relatively high risk of upper urinary tract cancers, prostate cancer, and brain tumors. Upper gastrointestinal cancers (gastric, small bowel, biliary and pancreatic) occurred from 40 years of age in *path_MLH1* and *path_MSH2* carriers*,* more frequently in males than females. The increased number of *path_PMS2* carriers in the expanded PLSD cohort allowed their stratification for the first time by gender, age and organ. They had modestly increased risks of late onset CRC (males: 32.8% [12.7–68.6] and females: 8.5% [2.1–31.5]) and endometrial cancer (21.2% [8.5–46.9]). No CRC or endometrial cancers were detected before 50 years in *path_PMS2* carriers.

Notably, a high risk of CRC or endometrial or ovarian cancer was observed for *path_MLH1* (79.7% [72.7–85.9]) and *path_MSH2* carriers (80.4% [72.2–87.5]) at 75 years of age and there was a 50% [39.1–62.9] lifetime risk for female *path_MSH6* carriers (Supplementary Tables S3–S6).

For carriers of a *path_MLH1, path_MSH2, path_MSH6* or *path_PMS2* variant, risks of CRC, gynaecological cancer and upper urinary tract cancer were similar regardless of whether they had a previous or prevalent cancer in other organs at inclusion for follow-up (Supplementary Table S8).

## Discussion

Most deaths following cancer in *path_MMR* carriers, particularly in women and in *path_MSH2* carriers of both genders, occurred after LS-associated cancers in organs other than the colorectum. While CRC incidence was high in those having colonoscopy surveillance, early diagnosis, treatment and the emergence of the immunotherapy^24^ probably contributed to the low CRC mortality observed. Any study like ours will inevitably have time-trend biases related to changes in treatment during the observation period. Because modern treatment may be associated with increased survival, our results are likely to represent minimum estimates for survival and maximum estimates for mortality. To further reduce mortality in LS, it may be reasonable to address prevention and treatment needs for the cancers now associated with most of the deaths.

We present cumulative cancer incidences for separate organs and groups of organs (Supplementary Tables S3–S6). If, as an example, we report the cumulative incidences in female *path_MLH1* carriers at 75 years, the cumulative incidences of colon and rectal cancer were 46.2% and 7.4%, respectively (the sum being 53.6%), but the cumulative incidence for colon *or* rectal cancer was 48.3%. The difference (5.3%) indicates how many carriers had both colon and rectal cancers. Similarly, for endometrial and ovarian cancer, the sum would be 45.2% while the observed value for endometrial *or* ovarian cancer was 43.2%. The sum of the cumulative incidences for all four cancers would be 98.8%, while the observed value was 79.7%. These observations reflect the interrelation between probabilities for having a cancer, depending on whether the individual has had another cancer or not, and also on the ages at which cancers in the various organs occur. For these reasons, the median ages at diagnosis of cancer in different organs by gene and gender, described here for the first time, will be of interest. We observed a higher incidence but not an earlier age at diagnosis for upper urinary tract cancers and prostate cancer in *path_MSH2* carriers compared to *path_MLH1, path_MSH6* and *path_PMS2* carriers. These observations appear consistent with the conclusions of a recent review from the European Association of Urology, Young Academic Urologists and the Global Society of Rare Genitourinary Tumors.^14^ The potential benefits of urological surveillance for patients with LS, particularly for *path_MSH2* carriers, merit further research.

Many of the issues related to cancer incidences are also relevant when considering mortality. Crude survival following cancer in one organ also depends on survival after any other synchronous or metachronous cancer that occurs, as well as other, non-cancer causes. Because the mortalities we observed were much lower than the cumulative incidence rates, the effects of these confounders were marginal for the survival values we calculated. If summing-up of the mortalities for cancer in each organ was performed, the sum would be artificially high, but not to the extent discussed above for cumulative incidence because of the low mortality rates. The mortality estimates that we present here for the first time are novel and robust estimates.

Obesity is associated with endometrial cancer in the general population, and it would be of interest to examine if this is so in *path_MMR* carriers as well. It would also be of interest to measure disease-specific survival. Both will need information so far not included in the PLSD dataset. In relation to the discussion on prophylactic hysterectomy and oophorectomy in LS, we have previously reported the limited effect of risk-reducing surgery on gynaecological cancer mortality in a smaller cohort.^9^ The small effects seen were in contrast to the much larger survival benefit achieved by oophorectomy in carriers of pathogenic variants in *BRCA1/2*.^25^

The current study, that includes close to three times as many observation years, confirmed the findings of our earlier report,^5^ that prospective cancer risks in *path_MMR* carriers are independent of the occurrence of previous or prevalent cancer, validating the use of our results for estimation of cancer risk in any organ in any carrier irrespective of their cancer history. Our website www.plsd.eu, which will be updated based upon the results in this report once it is published, enables such calculations to be made for individual *path_MMR* carriers, by age, gene and gender.

The strengths of this study include its prospective design and the substantial follow-up that has been accumulated. Its limitations include the lack of data on cancer-specific survival and the absence of a control group that has not been subjected to surveillance interventions. The extent to which the results obtained for CRC are attributable to early diagnosis and treatment as a result of colonoscopy surveillance, or to improved treatment over the decades during which the carriers were observed, is unknown.

The current study found low CRC mortality in *path_MMR* carriers who receive colonoscopy surveillance while some extracolonic cancers were associated with high mortality. Further improvement of survival in LS may require a focus on the prevention and treatment of non-colorectal cancers, likely including approaches based upon the immune response to MSI pre-cancerous lesions and cancers.^26^^,^^27^ This study also provides more precise cumulative cancer incidences for *path_MMR* carriers than have been available previously, stratified by age, gene, organ, and gender. Our interactive website www.PLSD.eu that is referred to by EHTG (www.ehtg.org) and the InSiGHT variant databases (http://insight-database.org/) will be updated to include the results of the current study following publication. The website enables interactive estimation of the remaining risk for any cancer in any *path_MMR* carrier who is receiving currently recommended CRC surveillance, by age, gene and gender.

## Contributors

P.M., M.D.-V., T.S.S and J.R.S designed the study. M.D.-V. is the PLSD curator and P.M. is the PI to the PLSD. M.D.-V., S.H. and P.M. calculated the results. M.D.-V., S.H., J.R.S., T.T.S. and P.M. wrote the manuscript. All authors contributed data to the PLSD and reviewed and approved the manuscript. All authors have read and agreed to the final version of the manuscript. PM has verified the submitted version. All authors had full access to all the data in the study and accept responsibility for the decision to submit for publication.

## Data sharing statement

The cancer risk algorithm is available at the PLSD website (www.plsd.eu) that is based upon the results presented in this report and enables interactive calculation of remaining lifetime risks for cancer in any patient with LS by giving their age, gender, and gene variant.

## Declaration of interests

R.B. has received honoraria for lectures and advisory boards from AbbVie, Amgen, AstraZeneca, Bayer, BMS, Boehringer-Ingelheim, Illumina, Lilly, Merck-Serono, MSD, Novartis, Qiagen, Pfizer, Roche, and Targos MP Inc. R.B. is a Co-Founder and Scientific Advisor for Targos Mol. Pathology Inc. Kassel/Germany. T.T.S. is the CEO and co-owner of Healthfund Finland Oy and reports consultation fees from Boehringer Ingelheim Finland and Amgen. FB is supported by JANSSEN PHARMACEUTICALS (clinical trial for Familial Adenomatous polyposis). RH is supported by the 10.13039/501100002424Fujifilm Germany and Janssen-Pharmaceuticals. LK is consultant of Sandoz, Novartis and Abbott. GM reports consultancy fees from Johnson & Johnson.
