# Supplementary material for: Mortality by age, gene and gender in carriers of pathogenic mismatch repair gene variants receiving surveillance for early cancer diagnosis and treatment: a report from the prospective Lynch syndrome database
Source: eClinicalMedicine. 2023 Mar 20;58:101909. doi: 10.1016/j.eclinm.2023.101909 (PMC10166779; doi:10.1016/j.eclinm.2023.101909)

Table S1. Patients included, follow-up years and age at inclusion stratified on pathogenic variants of the genes, gender and country.

Group	Gender	Cases	Follow-up years				Age at inclusion		
			Number	Mean	Min	Max	Mean	Min	Max
All		8500	71713	7.6	1	42	46.3	25	74
	F	4588	38102	7.5	1	41	46.5	25	74
	M	3912	33611	7.7	1	42	46.1	25	74
**Country**									
Denmark		1730	16854	9.7	1	37	44.3	25	74
Finland		1064	14267	13.4	1	38	42.0	25	74
Germany		998	6870	6.9	1	28	44.5	25	74
Australia		819	7219	8.8	1	33	45.6	25	74
Spain		694	3701	5.3	1	30	45.2	25	74
The Netherlands		524	3711	7.1	1	41	50.9	25	74
United Kingdom		598	4002	6.7	1	42	44.0	25	74
USA		385	2564	6.7	1	16	50.9	25	74
Italy		328	2653	8.1	1	33	40.8	25	74
Norway		296	2134	7.2	1	22	44.1	25	73
Israel		285	1594	5.6	1	37	44.2	25	73
Canada		180	1369	7.6	1	16	49.8	25	73
Sweden		155	1435	9.3	1	25	44.4	25	74
Switzerland		76	502	6.6	1	25	51.1	28	74
Uruguay		68	519	7.6	1	21	43.1	25	68
Poland		62	651	10.5	1	22	40.1	25	74
New Zealand		61	455	7.5	1	12	44.4	25	74
Brazil		55	473	8.6	1	26	45.5	25	66
Chile		44	269	6.1	1	13	43.9	25	66
Argentina		35	298	8.5	1	41	42.5	27	71
Ireland		18	74	4.1	1	15	48.2	28	64
Colombia		12	59	4.9	3	7	47.2	39	60
India		9	19	2.1	1	3	45.2	33	62
Mexico		3	15	5.0	3	7	28.3	25	35
Hungary		1	6	6.0	6	6	39.0	39	39
**Gene**	**Gender**	**Cases**	**Follow-up years**						
	F	1686	16586	9.8	1	38	43.3	25	74
MLH1	M	1445	14660	10.1	1	38	42.5	25	74
	F	1708	13976	8.2	1	41	44.6	25	74
MSH2	M	1463	12486	8.5	1	42	43.6	25	74
	F	884	5925	6.7	1	41	48.9	25	74
MSH6	M	765	5232	6.8	1	37	48.3	25	74
	F	310	1615	5.2	1	20	49.1	25	74
PMS2	M	239	1233	5.2	1	23	49.9	25	74

Table S2. Cancers detected during prospective observation in the combined dataset.

ICD9	Organ	N males	N females	N all	% of all cancers
141	Tongue	1	1	2	0,1
145	Mouth	2		2	0,1
150	Esophagus	7	3	10	0,5
151	Stomach	37	23	60	3,2
152	Small bowel	42	28	70	3,8
153	Colon	250	231	481	26,0
154	Rectum/sigmoid	72	65	137	7,4
155	Liver	6	5	11	0,6
156	Biliary tract	18	11	29	1,6
157	Pancreas	15	21	36	1,9
158	Peritoneum	1	2	3	0,2
159	Abdominal unspecified	3	1	4	0,2
160	Nasal cavity		1	1	0,1
161	Larynx	8	3	11	0,6
162	Lung	7	12	19	1,0
163	Pleura	1		1	0,1
164	Mediastinum	1	1	2	0,1
170	Osteosarcoma	5	6	11	0,6
171	Soft tissue sarcoma	3	1	4	0,2
172	Melanoma	9	7	16	0,9
173	Skin	80	75	155	8,4
174	Breast		108	108	5,8
179	Uterus unspecified		9	9	0,5
180	Cervix		8	8	0,4
182	Endometrium		237	237	12,8
183	Ovary		57	57	3,1
184	Female genital unspecified		7	7	0,4
185	Prostate	83		83	4,5
186	Testes	1		1	0,1
188	Urine bladder	46	37	83	4,5
189	Kidney/ureter	53	61	114	6,2
190	Eye	1		1	0,1
191	Brain	14	13	27	1,5
192	Nervous system unspecified	2		2	0,1
193	Thyroid	6	9	15	0,8
194	Adrenal gland	2	2	4	0,2
195	Unspecified	1	1	2	0,1
198	Spread from elsewhere		1	1	0,1
199	Unknown origin	12	5	17	0,9
200	Non-Hodking lymphoma	3	1	4	0,2
201	Hodking		1	1	0,1
202	Lymphoma others		1	1	0,1
203	Multiple myeloma	1		1	0,1
204	Acute lymphatic leukemia/chronic lymphatic leukemia		1	1	0,1
208	Leukemia unspecified	3	1	4	0,2

Table S3. Cumulative incidences with [95% confidence intervals]in respective organs by age and gender for *path_MLH1* carriers.

Site	Cumulative incidence 30 years		Cumulative incidence 40 years		Cumulative incidence 50 years		Cumulative incidence 60 years		Cumulative incidence 70 years		Cumulative incidence 75 years	
	Males	Females	Males	Females	Males	Females	Males	Females	Males	Females	Males	Females
Any organ	3.3 [1.5-7.1]	0 [-]	16.7 [12.7-21.9]	12.4 [9.1-16.7]	37.8 [32.5-43.7]	36.8 [31.9-42.3]	54.5 [48.5-60.7]	59.5 [53.9-65.1]	63.0 [56.1-69.8]	75.5 [69.4-81.3]	68.5 [60.8-76.0]	80.2 [73.5-86.1]
Colon*	2.7 [1.1-6.3]	0 [-]	14.0 [10.3-18.8]	8.1 [5.6-11.7]	32.0 [27.0-37.6]	16.3 [12.8-20.6]	45.4 [39.7-51.6]	28.6 [24.0-34.0]	53.0 [46.4-60.0]	40.0 [34.3-46.4]	55.9 [48.6-63.4]	46.2 [39.3-53.7]
Sigmoid/Rectum*	0.5 [0.1-3.5]	0 [-]	1.6 [0.6-3.9]	1.1 [0.4-3]	2.8 [1.5-5.3]	2.0 [1.0-4.0]	4.7 [2.9-7.6]	3.9 [2.4-6.4]	7.8 [5.1-11.8]	6.0 [3.8-9.3]	10.7 [6.9-16.3]	7.4 [4.7-11.4]
Colorectal*	3.3 [1.5-7.1]	0 [-]	14.9 [11.1-19.9]	9.0 [6.3-12.8]	32.5 [27.4-38.3]	18.2 [14.5-22.8]	45.9 [40.1-52.3]	30.6 [25.8-36.1]	52.8 [46.1-59.8]	42.1 [36.2-48.6]	56.0 [48.5-63.7]	48.3 [41.3-55.9]
Endometrium		0 [-]		2.0 [1.0-4.2]		14.9 [11.6-19.1]		28.4 [23.6-33.9]		35.8 [29.9-42.5]		37.2 [30.9-44.3]
Ovary		0 [-]		1.4 [0.6-3.5]		4.4 [2.7-7.2]		7.4 [4.9-11.1]		8.0 [5.3-12.0]		8.0 [5.3-12.0]
Endometrium/ ovary		0 [-]		3.4 [1.9-5.9]		18.8 [15.1-23.3]		34.5 [29.4-40.1]		41.9 [36.0-48.5]		43.2 [36.9-50.0]
CRC/Endometriu m/ovary cancer		0 [-]		12.5 [9.3-16.8]		35.4 [30.4-41.1]		60.5 [54.4-66.6]		75.0 [68.4-81.1]		79.7 [72.7-85.9]
Urine bladder	0 [-]	0 [-]	0 [-]	0 [-]	0.5 [0.1-1.9]	0.2 [0-1.5]	2.1 [1.0-4.3]	1.0 [0.4-2.7]	3.8 [2.1-6.9]	2.7 [1.3-5.5]	5.6 [3.1-10.0]	4.8 [2.6-8.9]
Ureter/kidney	0 [-]	0 [-]	0 [-]	0.3 [0-1.8]	0.7 [0.2-2.2]	0.5 [0.1-1.9]	1.3 [0.5-3.2]	0.7 [0.2-2.2]	3.7 [1.9-6.9]	2.2 [1.0-4.6]	4.5 [2.4-8.6]	2.9 [1.4-6.0]
Kidney, ureter and/or urine bladder	0 [-]	0 [-]	0 [-]	0.3 [0-1.8]	1.2 [0.5-2.9]	0.7 [0.2-2.1]	3.4 [1.9-5.9]	1.7 [0.8-3.6]	6.5 [4.1-10.3]	4.1 [2.4-7.1]	9.2 [5.8-14.2]	6.9 [4.1-11.3]
Kidney, ureter and/or urine bladder/prostate	0 [-]		0 [-]		1.4 [0.7-3.2]		6.0 [3.9-9.1]		13.3 [9.7-18.3]		23.9 [17.9-31.4]	
Gastric	0 [-]	0 [-]	0 [-]	0.3 [0-1.8]	0.7 [0.2-2.2]	0.5 [0.1-1.9]	2.4 [1.2-4.6]	1.0 [0.4-2.7]	5.5 [3.3-9.3]	2.9 [1.5-5.6]	8.9 [5.5-14.4]	4.3 [2.3-7.9]
Small bowel	0 [-]	0 [-]	0.3 [0-1.9]	0.9 [0.3-2.8]	1.5 [0.7-3.2]	1.6 [0.7-3.5]	3.6 [2.1-6.1]	2.5 [1.3-4.6]	6.6 [4.1-10.3]	3.8 [2.2-6.7]	8.3 [5.2-13.0]	4.5 [2.6-7.8]
Pancreas	0 [-]	0 [-]	0.3 [0-1.9]	0.3 [0-2.3]	0.5 [0.1-2.0]	0.8 [0.2-2.4]	1.1 [0.4-2.9]	1.2 [0.5-2.9]	2.2 [0.9-5.1]	2.3 [1.1-4.7]	3.1 [1.4-6.9]	3.7 [1.9-7.2]
Bile duct/gall bladder	0 [-]	0 [-]	0 [-]	0 [-]	0.7 [0.2-2.2]	0.4 [0.1-1.7]	1.3 [0.5-3.1]	1.2 [0.5-2.8]	4.0 [2.2-7.3]	1.5 [0.7-3.3]	4.0 [2.2-7.3]	1.5 [0.7-3.3]
Prostate	0 [-]		0 [-]		0.2 [0-1.7]		2.9 [1.5-5.4]		7.6 [4.8-11.8]		15.6 [10.7-22.6]	
Breast		0 [-]		0.6 [0.1-2.3]		3.0 [1.7-5.1]		7.4 [5.3-10.4]		10.4 [7.6-14.1]		12.4 [9.1-16.9]
Brain	0 [-]	0 [-]	0 [-]	0.3 [0-1.8]	0 [-]	0.7 [0.2-2.1]	0 [-]	0.9 [0.3-2.4]	0.6 [0.1-3.9]	1.4 [0.5-3.4]	0.6 [0.1-3.9]	1.4 [0.5-3.4]

Table S4. Cumulative incidences with [95% confidence intervals] in respective organs by age and gender for *path_MSH2* carriers.

Site	Cumulative incidence 30 years		Cumulative incidence 40 years		Cumulative incidence 50 years		Cumulative incidence 60 years		Cumulative incidence 70 years		Cumulative incidence 75 years	
	Males	Females	Males	Females	Males	Females	Males	Females	Males	Females	Males	Females
Any organ	3.6 [1.5-8.4]	2.3 [0.9-6.1]	12.7 [8.7-18.3]	15.1 [11.0-20.7]	31.1 [25.5-37.8]	40.1 [34.2-46.6]	59.4 [52.4-66.5]	58.8 [52.6-65.2]	73.2 [65.5-80.4]	77.7 [71.0-83.8]	80.5 [72.3-87.5]	83.4 [76.8-89.1]
Colon*	3.4 [1.4-8.0]	1.7 [0.5-5.1]	8.4 [5.2-13.3]	7.1 [4.4-11.3]	19.9 [15.3-25.7]	14.9 [11.1-19.8]	34.2 [28.3-40.9]	22.1 [17.7-27.5]	47.3 [39.9-55.2]	36.5 [30.5-43.2]	52.7 [44.4-61.4]	38.4 [32.1-45.5]
Sigmoid/Rectum*	0 [-]	0 [-]	1.3 [0.4-3.9]	0.3 [0-2.5]	5.8 [3.7-9.1]	2.4 [1.2-4.8]	10.6 [7.6-14.8]	5.0 [3.2-8.0]	14.7 [10.8-20.0]	12.2 [8.7-16.9]	18.2 [13.1-24.9]	13.7 [9.9-19.0]
Colorectal*	3.6 [1.5-8.4]	1.7 [0.6-5.2]	10.3 [6.7-15.6]	7.3 [4.5-11.6]	21.7 [16.8-27.9]	15.8 [11.8-21.0]	38.6 [32.0-45.9]	24.2 [19.4-29.9]	51.0 [42.8-59.7]	39.8 [33.5-46.7]	55.8 [46.8-65.1]	42.6 [35.9-49.9]
Endometrium		0 [-]		2.0 [0.8-4.8]		18.1 [13.9-23.5]		33.9 [28.0-40.5]		42.1 [35.0-50.1]		44.1 [36.3-52.7]
Ovary		0 [-]		1.6 [0.6-4.2]		8.3 [5.5-12.4]		9.7 [6.6-14.2]		13.4 [8.9-20.1]		13.4 [8.9-20.1]
Endometrium/ ovary		0 [-]		3.6 [1.9-6.8]		25.7 [20.7-31.5]		41.6 [35.6-48.2]		51.5 [44.4-59.1]		53.3 [45.6-61.3]
CRC/Endometrium/ ovary cancer		1.7 [0.6-5.3]		10.9 [7.4-15.9]		37.6 [31.5-44.5]		55.8 [48.9-62.8]		75.1 [67.7-81.9]		80.4 [72.2-87.5]
Urine bladder	0 [-]	0 [-]	0 [-]	0.4 [0.1-3.1]	1.1 [0.4-3.0]	1.5 [0.6-3.7]	4.6 [2.7-7.6]	2.6 [1.3-4.9]	7.2 [4.6-11.3]	6.2 [3.9-9.8]	13.1 [8.3-20.3]	9.4 [6.0-14.5]
Ureter/kidney	0 [-]	0 [-]	0 [-]	0 [-]	1.7 [0.8-3.7]	1.9 [0.9-4.0]	6.8 [4.5-10.2]	5.1 [3.3-7.9]	13.4 [9.7-18.5]	13.9 [10.2-18.6]	15.8 [11.2-22.0]	19.5 [14.6-25.9]
Kidney, ureter and/or urine bladder	0 [-]	0 [-]	0 [-]	0.4 [0.1-3.1]	2.5 [1.3-4.8]	2.6 [1.4-5.1]	9.8 [7.0-13.7]	6.4 [4.3-9.4]	18.1 [13.7-23.6]	17.4 [13.3-22.6]	25.1 [18.9-33.0]	23.7 [18.4-30.3]
Kidney, ureter and/or urine bladder/prostate	0 [-]		0 [-]		3.1 [1.7-5.5]		15.6 [12.0-20.2]		31.7 [25.9-38.4]		39.7 [32.4-48.0]	
Gastric	0 [-]	0 [-]	0 [-]	0 [-]	0,3 [0-1.9]	0.5 [0.1-2.1]	3.0 [1.6-5.7]	1.6 [0.7-3.5]	5.0 [2.9-8.5]	3.1 [1.7-5.9]	8.3 [4.8-14.2]	4.0 [2.1-7.4]
Small bowel	0 [-]	0 [-]	0.7 [0.2-2.6]	0 [-]	0.9 [0.3-2.9]	1.4 [0.6-3.3]	3.6 [2.0-6.4]	2.5 [1.3-4.7]	7.0 [4.3-11.3]	3.7 [2.1-6.4]	7.0 [4.3-11.3]	3.7 [2.1-6.4]
Pancreas	0 [-]	0 [-]	0 [-]	0 [-]	0 [-]	0.3 [0-1.9]	0.9 [0.3-2.9]	0.5 [0.1-2.1]	3.3 [1.5-7.1]	2.7 [1.3-5.8]	3.3 [1.5-7.1]	3.5 [1.7-7.3]
Bile duct/gall bladder	0 [-]	0 [-]	0 [-]	0 [-]	0.3 [0-1.9]	0 [-]	0.6 [0.1-2.2]	0.8 [0.3-2.4]	2.3 [0.9-5.7]	0.8 [0.3-2.4]	4.6 [2.0-10.2]	2.4 [0.9-6.5]
Prostate	0 [-]		0 [-]		0.8 [0.3-2.5]		6.6 [4.4-10.0]		16.4 [12.0-22.1]		24.0 [17.8-32.0]	
Breast		0 [-]		1.2 [0.4-3.7]		3.4 [1.9-6.1]		7.4 [5.1-10.7]		13.2 [9.7-17.7]		15.5 [11.5-20.9]
Brain	0 [-]	0 [-]	0.4 [0.1-3.0]	0.9 [0.2-3.5]	1.3 [0.5-3.5]	0.9 [0.2-3.5]	1.9 [0.9-4.3]	1.4 [0.5-3.8]	3.3 [1.7-6.3]	1.4 [0.5-3.8]	6.6 [3.4-12.4]	2.2 [0.8-5.7]

Table S5. Cumulative incidences with [95% confidence intervals] in respective organs by age and gender for *path_MSH6* carriers.

Site	Cumulative incidence 30 years		Cumulative incidence 40 years		Cumulative incidence 50 years		Cumulative incidence 60 years		Cumulative incidence 70 years		Cumulative incidence 75 years	
	Males	Females	Males	Females	Males	Females	Males	Females	Males	Females	Males	Females
Any organ	2.7 [0.4-17.7]	0 [-]	5.9 [1.9-17.7]	1.3 [0.2-8.7]	9.4 [4.2-20.5]	13.7 [8.4-22.1]	20.7 [12.9-32.3]	33.0 [24.8-43.1]	22.6 [14.3-34.5]	52.1 [40.6-64.7]	28.5 [18.3-42.6]	55.2 [43.0-68.2]
Colon*	2.6 [0.4-17.1]	0 [-]	5.5 [1.7-16.8]	1.1 [0.2-7.5]	6.5 [2.3-17.3]	4.5 [1.9-10.5]	11.5 [5.9-21.7]	7.4 [3.9-13.7]	12.7 [6.8-23.1]	15.5 [9.9-24]	17.1 [9.7-29.2]	15.5 [9.9-24]
Sigmoid/Rectum*	0 [-]	0 [-]	0 [-]	0 [-]	0 [-]	0 [-]	2.4 [0.8-7.3]	1.9 [0.6-5.6]	6.2 [3.0-12.7]	4.7 [2.3-9.7]	6.2 [3.0-12.7]	4.7 [2.3-9.7]
Colorectal*	2.7 [0.4-17.7]	0 [-]	5.8 [1.8-17.6]	1.2 [0.2-8.1]	6.9 [2.5-18.3]	4.0 [1.5-10.3]	11.8 [5.9-22.8]	8.6 [4.7-15.5]	13.5 [7.1-24.8]	17.3 [11.2-26.3]	16.4 [8.8-29.4]	17.3 [11.2-26.3]
Endometrium		0 [-]		1.1 [0.2-7.4]		8.6 [4.7-15.4]		22.8 [16.3-31.5]		41.4 [32.3-52.0]		45.7 [35.6-57.0]
Ovary		0 [-]		1.1 [0.2-7.4]		1.9 [0.5-7.5]		2.9 [0.9-8.7]		6.3 [2.6-15.2]		6.3 [2.6-15.2]
Endometrium/ ovary		0 [-]		2.2 [0.5-8.3]		10.3 [6.0-17.5]		25.1 [18.2-33.9]		45.7 [36.3-56.1]		49.6 [39.5-60.6]
CRC/Endometrium/ ovary cancer		0 [-]		3.7 [1.2-10.9]		15.6 [9.7-24.6]		29.2 [21.2-39.5]		50.4 [39.1-62.9]		50.4 [39.1-62.9]
Urine bladder	0 [-]	0 [-]	0 [-]	0 [-]	0.8 [0.1-5.6]	0 [-]	1.5 [0.4-5.8]	1.2 [0.3-4.5]	3.0 [1.1-7.9]	2.6 [1.0-6.8]	9.0 [4.7-16.9]	2.6 [1.0-6.8]
Ureter/kidney	0 [-]	0 [-]	0 [-]	0 [-]	0 [-]	0 [-]	1.4 [0.3-5.4]	1.2 [0.3-4.6]	3.3 [1.2-8.7]	3.9 [1.8-8.6]	3.3 [1.2-8.7]	3.9 [1.8-8.6]
Kidney, ureter and/or urine bladder	0 [-]	0 [-]	0 [-]	0 [-]	0.8 [0.1-5.6]	0 [-]	2.2 [0.7-6.6]	2.3 [0.9-6.1]	4.7 [2.1-10.2]	6.5 [3.5-11.8]	10.9 [6.0-19.1]	6.5 [3.5-11.8]
Kidney, ureter and/or urine bladder/prostate	0 [-]		0 [-]		0.8 [0.1-5.6]		3.6 [1.5-8.5]		8.9 [5.0-15.7]		16.4 [10.4-25.4]	
Gastric	0 [-]	0 [-]	0 [-]	0 [-]	0 [-]	0 [-]	0.7 [0.1-4.9]	0 [-]	0.7 [0.1-4.9]	0.7 [0.1-4.7]	0.7 [0.1-4.9]	0.7 [0.1-4.7]
Small bowel	0 [-]	0 [-]	0 [-]	0 [-]	0 [-]	0 [-]	0.7 [0.1-4.8]	0.6 [0.1-4.0]	1.6 [0.4-6.5]	0.6 [0.1-4.0]	2.8 [0.9-8.7]	0.6 [0.1-4.0]
Pancreas	0 [-]	0 [-]	0 [-]	0 [-]	0 [-]	0.7 [0.1-4.8]	0 [-]	0.7 [0.1-4.8]	0 [-]	2.2 [0.7-6.8]	1.2 [0.2-8.1]	2.2 [0.7-6.8]
Bile duct/gall bladder	0 [-]	0 [-]	0 [-]	0 [-]	0 [-]	0 [-]	0 [-]	0 [-]	0 [-]	0 [-]	0 [-]	0 [-]
Prostate	0 [-]		0 [-]		0 [-]		1.4 [0.4-5.5]		5.8 [2.8-11.8]		7.0 [3.5-13.7]	
Breast		0 [-]		0 [-]		2.3 [0.8-7.1]		6.5 [3.5-11.7]		9.3 [5.6-15.3]		15.1 [9.7-23.1]
Brain	0 [-]	0 [-]	0 [-]	0 [-]	0 [-]	0 [-]	0 [-]	1.2 [0.3-4.6]	0.8 [0.1-5.3]	1.2 [0.3-4.6]	0.8 [0.1-5.3]	1.2 [0.3-4.6]

Table S6. Cumulative incidences with [95% confidence intervals] in respective organs by age and gender for *path_PMS2* carriers.

Site	Cumulative incidence 30 years		Cumulative incidence 40 years		Cumulative incidence 50 years		Cumulative incidence 60 years		Cumulative incidence 70 years		Cumulative incidence 75 years	
	Males	Females	Males	Females	Males	Females	Males	Females	Males	Females	Males	Females
Any organ	0 [-]	0 [-]	0 [-]	0 [-]	6.7 [1.0-38.7]	4.3 [0.6-26.8]	30.1 [14.8-55.1]	17.4 [7.6-36.9]	30.1 [14.8-55.1]	20.9 [10.0-40.7]	57.3 [33.4-83.1]	40.1 [19.9-69.3]
Colon*	0 [-]	0 [-]	0 [-]	0 [-]	0 [-]	0 [-]	9.5 [2.5-32.9]	0 [-]	15.9 [5.3-42.2]	7.7 [1.9-28.9]	35.4 [15.5-67.7]	7.7 [1.9-28.9]
Sigmoid/Rectum*	0 [-]	0 [-]	0 [-]	0 [-]	0 [-]	0 [-]	0 [-]	0 [-]	0 [-]	2.2 [0.3-14.6]	0 [-]	2.2 [0.3-14.6]
Colorectal*	0 [-]	0 [-]	0 [-]	0 [-]	0 [-]	0 [-]	10.5 [2.7-36.0]	0 [-]	10.5 [2.7-36.0]	8.5 [2.1-31.5]	32.8 [12.7-68.6]	8.5 [2.1-31.5]
Endometrium		0 [-]		0 [-]		0 [-]		9.9 [3.8-24.3]		12.7 [5.5-27.9]		21.2 [8.5-46.9]
Ovary		0 [-]		0 [-]		0 [-]		2.5 [0.4-16.3]		2.5 [0.4-16.3]		2.5 [0.4-16.3]
Endometrium/ ovary		0 [-]		0 [-]		0 [-]		12.4 [5.3-27.2]		15.1 [7.1-30.5]		23.3 [10.2-48.0]
CRC/Endometrium/ ovary cancer		0 [-]		0 [-]		0 [-]		10.3 [3.4-28.6]		14.5 [5.7-34.3]		28.0 [10.1-63.9]
Urine bladder	0 [-]	0 [-]	0 [-]	0 [-]	0 [-]	0 [-]	0 [-]	0 [-]	0 [-]	0 [-]	0 [-]	0 [-]
Ureter/kidney	0 [-]	0 [-]	0 [-]	0 [-]	0 [-]	0 [-]	0 [-]	0 [-]	0 [-]	0 [-]	5.1 [0.7-30.9]	0 [-]
Kidney, ureter and/or urine bladder	0 [-]	0 [-]	0 [-]	0 [-]	0 [-]	0 [-]	0 [-]	0 [-]	0 [-]	0 [-]	5.1 [0.7-30.9]	0 [-]
Kidney, ureter and/or urine bladder/prostate	0 [-]		0 [-]		3.3 [0.5-21.5]		3.3 [0.5-21.5]		3.3 [0.5-21.5]		8.6 [2.1-31.1]	
Gastric	0 [-]	0 [-]	0 [-]	0 [-]	0 [-]	0 [-]	2.7 [0.4-17.5]	0 [-]	2.7 [0.4-17.5]	0 [-]	2.7 [0.4-17.5]	0 [-]
Small bowel	0 [-]	0 [-]	0 [-]	0 [-]	3.3 [0.5-21.3]	0 [-]	3.3 [0.5-21.3]	0 [-]	3.3 [0.5-21.3]	2.1 [0.3-14.0]	3.3 [0.5-21.3]	2.1 [0.3-14.0]
Pancreas	0 [-]	0 [-]	0 [-]	0 [-]	0 [-]	0 [-]	0 [-]	0 [-]	0 [-]	0 [-]	0 [-]	0 [-]
Bile duct/gall bladder	0 [-]	0 [-]	0 [-]	0 [-]	0 [-]	0 [-]	0 [-]	0 [-]	0 [-]	0 [-]	0 [-]	0 [-]
Prostate	0 [-]		0 [-]		3.3 [0.5-21.5]		3.3 [0.5-21.5]		3.3 [0.5-21.5]		3.3 [0.5-21.5]	
Breast		0 [-]		0 [-]		0 [-]		6.0 [2.0-17.5]		6.0 [2.0-17.5]		12.4 [4.0-34.7]
Brain	0 [-]	7.3 [1.1-41.6]	0 [-]	7.3 [1.1-41.6]	0 [-]	7.3 [1.1-41.6]	0 [-]	7.3 [1.1-41.6]	0 [-]	7.3 [1.1-41.6]	0 [-]	7.3 [1.1-41.6]

Incidence at when 25 years set to zero. Cumulative incidences during age periods given.

*Following intervention with surveillance colonoscopy and gyneacological examinations as described in text.

Table S7. Crude survival (%) after selected cancers diagnosed after initiation of colonoscopy surveillance and before age of 65 years for path_MLH1, path_MSH2 and path_MSH6 carriers.

Cancer	Organ	n	5-year survival (%)	95% CI (%)	10-year survival (%)	95% CI (%)
Colorectal						
	Colon	428	94	[91-96]	87	[82-91]
	Rectal and sigmoid	107	78	[70-88]	72	[61-84]
Gynaecological						
	Endometrium	211	94	[91-98]	92	[88-96]
	Ovarian	50	90	[82-100]	85	[75-97]
Urinary tract						
	Ureter and kidney	85	86	[79-95]	73	[61-87]
	Urinary bladder	54	88	[78-99]	71	[57-89]
Others						
	Prostate	49	92	[84-100]	76	[61-94]
	Stomach	40	63	[48-81]	63	[48-81]
	Small bowel	51	80	[69-93]	70	[56-89]
	Biliary tract	21	50	[32-78]	42	[24-74]
	Pancreas	20	17	[5-57]	17	[5-57]
	Brain	20	34	[18-65]	34	[18-65]

Table S8. Median age of onset and cumulative incidences with [95% confidence intervals] of *path_MMR* carriers not having had previous or prevalent cancers at inclusion by group of organs, age, and gender.

Site	Median age of onset		Cumulative incidence 30 years		Cumulative incidence 40 years		Cumulative incidence 50 years		Cumulative incidence 60 years		Cumulative incidence 70 years		Cumulative incidence 75 years	
	Males	Females	Males	Females	Males	Females	Males	Females	Males	Females	Males	Females	Males	Females
Path_MLH1														
Colorectal*	46.9 [43.9 -49.8]	56.9 [53.0-60.9]	3.3 [1.5-7.1]	0 [-]	15.0 [11.2-20.0]	9.0 [6.3-12.9]	32.8 [27.7-38.7]	17.7 [13.9-22.4]	46.8 [40.8-53.2]	30.6 [25.4-36.6]	52.9 [46.0-60.1]	44.1 [36.9-52.1]	54.8 [47.3-62.6]	53.2 [43.3-63.8]
Endometrium /ovary		51.3 [48.9-53.5]		0 [-]		3.4 [1.9-6.3]		19.3 [15.0-24.6]		35.4 [29.1-42.7]		41.2 [33.4-50.1]		45.1 [35.2-56.5]
Kidney, ureter and/or urine bladder	70.9 [NA]	66.5 [NA]	0 [-]	0 [-]	0 [-]	0.3 [0-2.2]	1.3 [0.4-3.9]	0.7 [0.2-2.6]	1.3 [0.4-3.9]	0.7 [0.2-2.6]	2.3 [0.8-6.7]	2.3 [0.5-9.2]	6.0 [1.7-19.9]	2.3 [0.5-9.2]
Path_MSH2														
Colorectal*	53.0 [47.8 -56.8]	59.4 [50.1-63.4]	3.6 [1.5-8.4]	1.7 [0.6-5.3]	10.0 [6.4-15.3]	7.5 [4.7-12.0]	22.1 [17.0-28.4]	16.9 [12.5-22.5]	38.6 [31.7-46.4]	22.9 [17.6-29.4]	46.9 [38.7-55.9]	40.8 [32.2-50.8]	52.4 [42.3-63.3]	45.2 [35.5-56.2]
Endometrium /ovary		51.5 [49.0-54.0]		0 [-]		3.3 [1.6-6.8]		23.3 [17.7-30.3]		40.4 [32.9-48.8]		50.3 [40.9-60.7]		54.7 [43.1-67.2]
Kidney, ureter and/or urine bladder	62.5 [57.8 -70.7]	66.8 [61.5-72.4]	0 [-]	0 [-]	0 [-]	0.5 [0.1-3.4]	0.5 [0.1-3.3]	1.8 [0.7-4.8]	9.3 [5.2-16.3]	4.5 [2.2-8.8]	19.0 [11.3-31.0]	15.4 [8.9-26.1]	23.2 [13.5-38.2]	21.1 [12.4-34.6]
Path_MSH6														
Colorectal*	55.6 [NA-73.4]	62.6 [NA]	2.7 [0.4-17.7]	0 [-]	5.9 [1.9-17.7]	1.3 [0.2-8.7]	7.0 [2.6-18.5]	3.4 [1.1-10.1]	12.3 [6.2-23.5]	5.8 [2.4-13.4]	14.2 [7.5-26.0]	13.6 [6.6-27.2]	17.5 [9.4-31.3]	13.6 [6.6-27.2]
Endometrium /ovary		56.9 [49.8- 63.1]		0 [-]		1.3 [0.2-8.8]		11.0 [6.0-19.4]		24.0 [16.3-34.6]		38.1 [26.9-52.1]		38.1 [26.9-52.1]
Kidney, ureter and/or urine bladder	70.7 [NA]	61.3 [NA]	0 [-]	0 [-]	0 [-]	0 [-]	1.3 [0.2-8.6]	0 [-]	2.6 [0.7-10.2]	0.9 [0.1-6.4]	2.6 [0.7-10.2]	3.0 [0.7-12.5]	6.4 [1.8-21.5]	3.0 [0.7-12.5]
Path_PMS2														
Colorectal*	71.6 [NA]	62.5 [NA]	0 [-]	0 [-]	0 [-]	0 [-]	0 [-]	0 [-]	10.5 [2.7-36.0]	0 [-]	10.5 [2.7-36.0]	3.7 [0.5-23.7]	38.2 [14.6-76.9]	3.7 [0.5-23.7]
Endometrium /ovary		70. [NA]		0 [-]		0 [-]		0 [-]		10.9 [3.7-30.1]		10.9 [3.7-30.1]		26.0 [8.0-66.2]
Kidney, ureter and/or urine bladder	NA	NA	NA	0 [-]	NA	0 [-]	NA	0 [-]	NA	0 [-]	NA	0 [-]	NA	0 [-]

* Following intervention with surveillance colonoscopy and gyneacological examinations as described in text.; NA: occurs for the median age of onset or the confidence limits if the corresponding conditional cumulative incidence estimates always are above or below 50%.

Figure S1. Survival following rectal cancer by pathogenic variants of the gene.

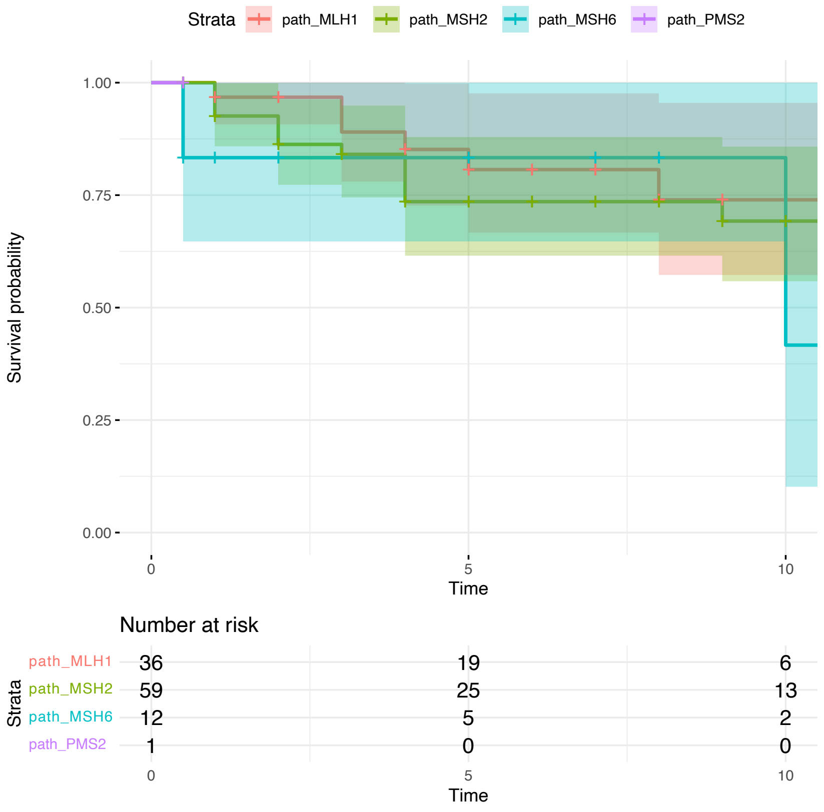

Figure S2. Survival following ovarian cancer by pathogenic variants of the gene.

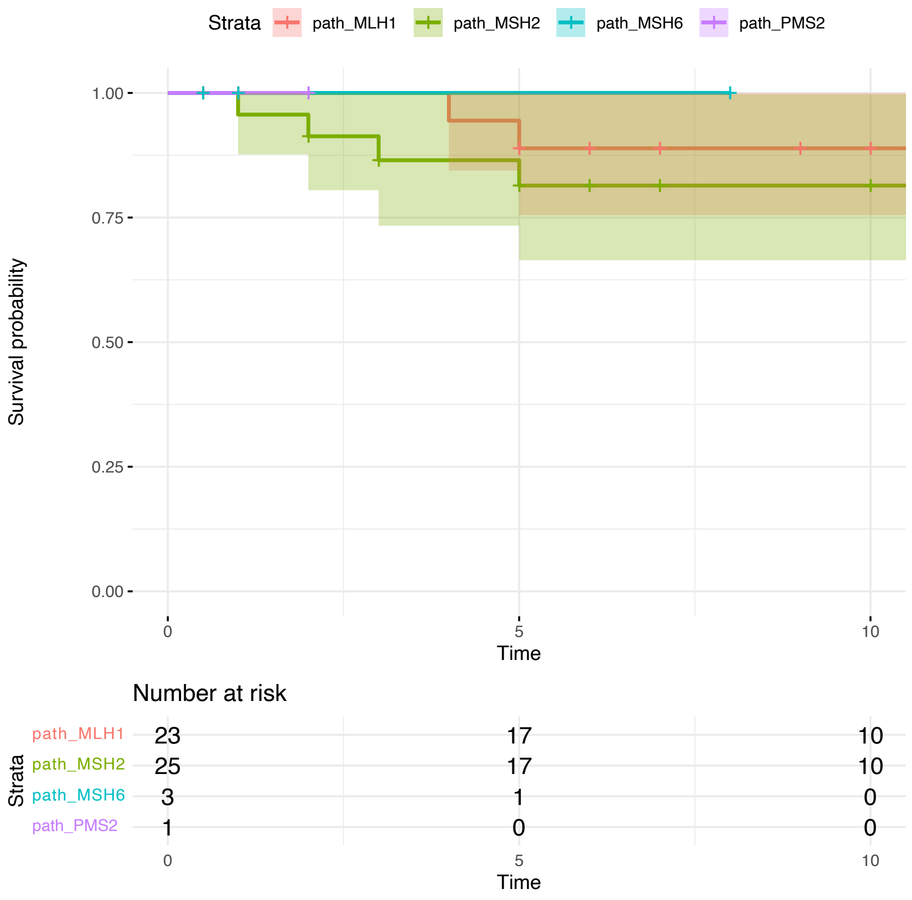

Figure S3. Survival following ureter and kidney cancer by pathogenic variants of the gene.

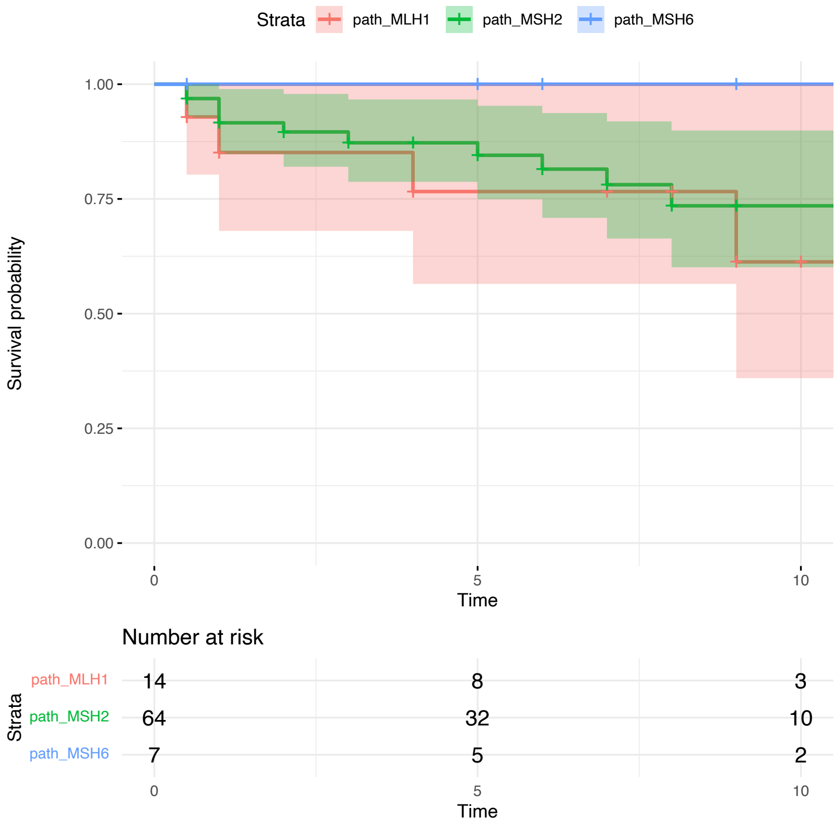

Figure S4. Survival following urinary bladder cancer by pathogenic variants of the gene.

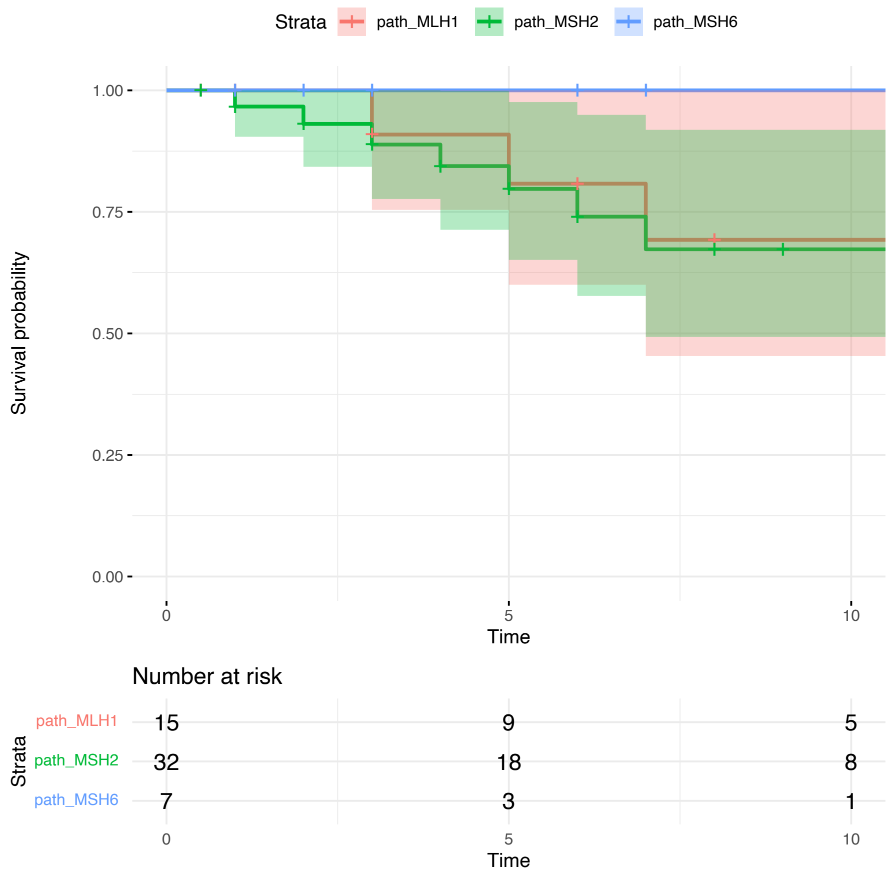

Figure S5. Survival following prostate cancer by pathogenic variants of the gene.

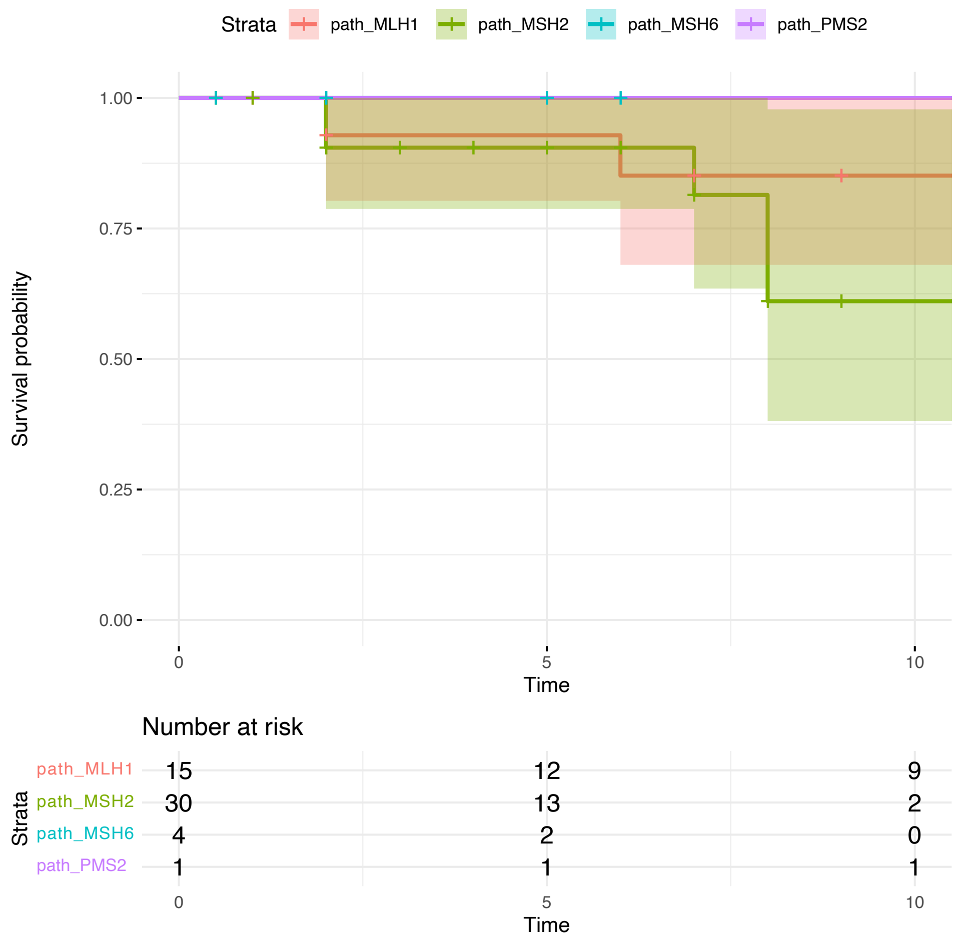

Figure S6. Survival following stomach cancer by pathogenic variants of the gene.

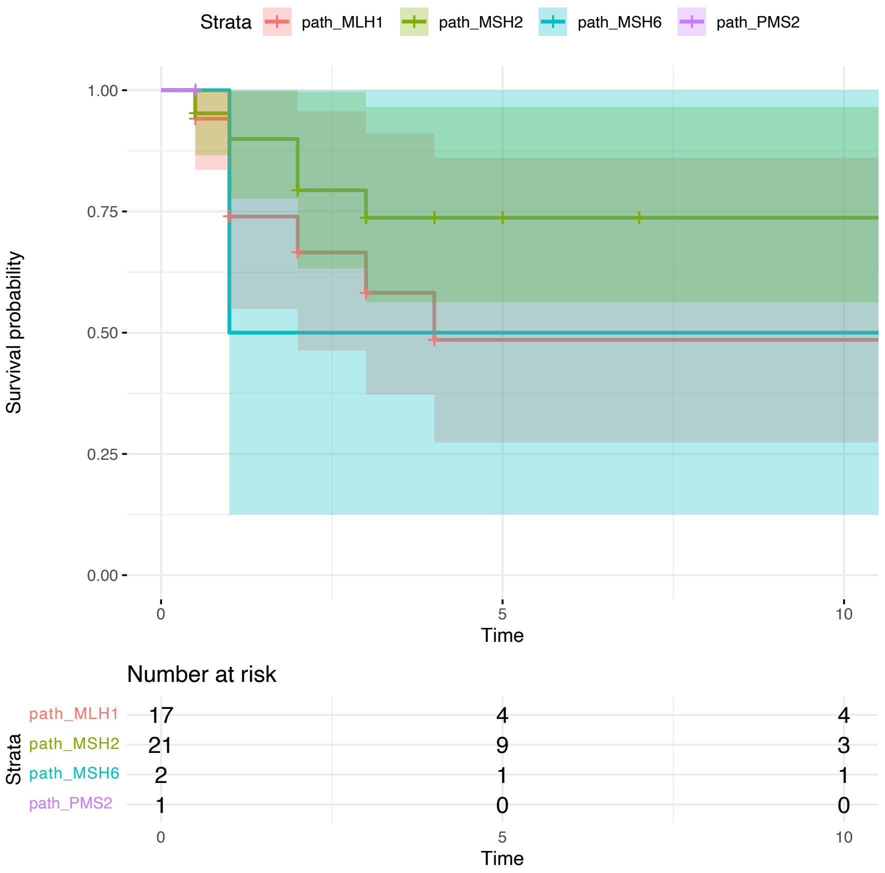

Figure S7. Survival following small bowel cancer by pathogenic variants of the gene.

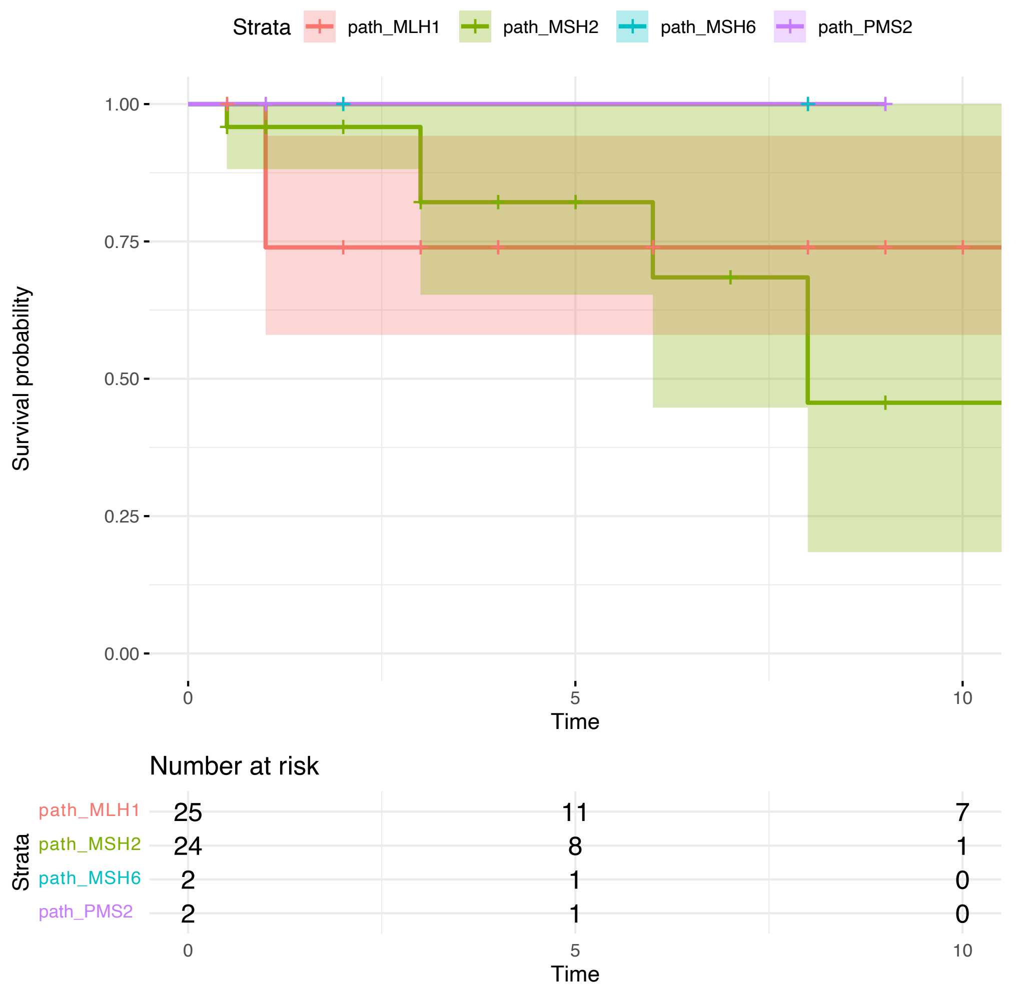

Figure S8. Survival following biliary tract cancer by pathogenic variants of the gene.

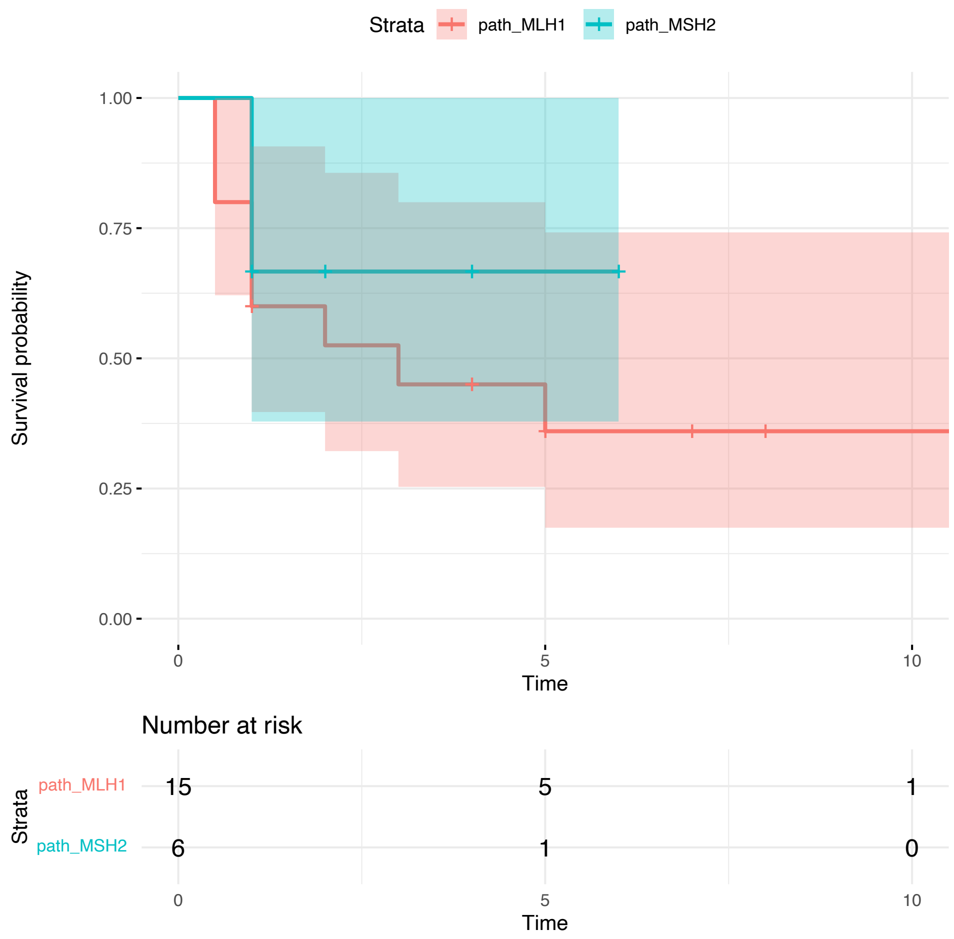

Figure S9. Survival following pancreas cancer by pathogenic variants of the gene.

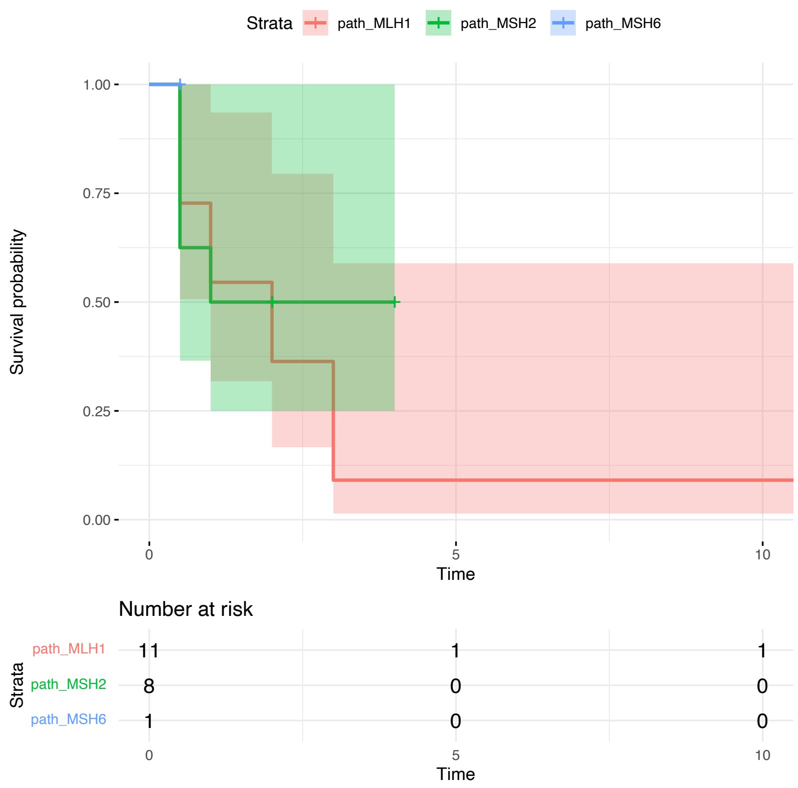

Strata path_MLH1 path_MSH2 path_MSH6
Survival probability
1.00
0.75
0.50
0.25
0.00
0
5
10
Time
Number at risk
Strata
path_MLH1 11 1 1
path_MSH2 8 0 0
path_MSH6 1 0 0
0
5
10
Time

Figure S10. Survival following brain cancer by pathogenic variants of the gene.

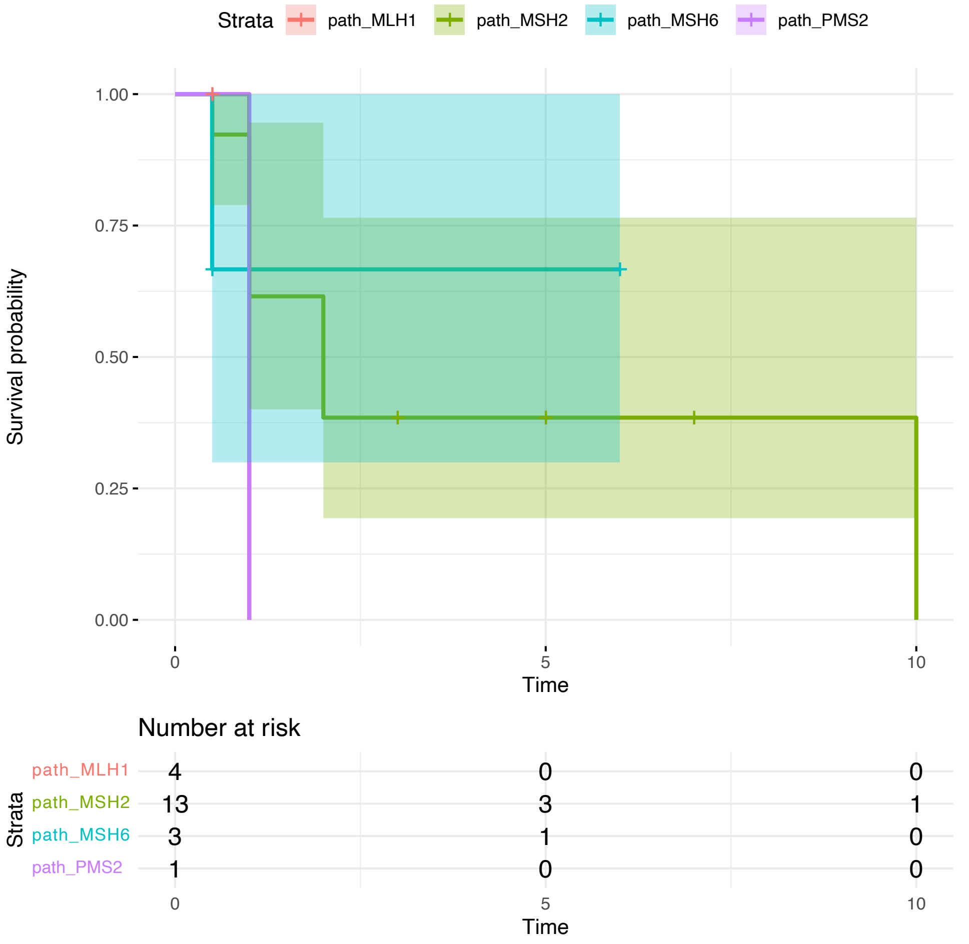

Supplement: Supplementary Figs. S1–S10 and Tables S1–S8 [file mmc1.pdf]
